# Exploring the role of *E. faecalis* enterococcal polysaccharide antigen (EPA) and lipoproteins in evasion of phagocytosis

**DOI:** 10.1111/mmi.15294

**Published:** 2024-07-12

**Authors:** Joshua S. Norwood, Jessica L. Davis, Bartłomiej Salamaga, Charlotte E. Moss, Simon A. Johnston, Philip M. Elks, Endre Kiss-Toth, Stéphane Mesnage

**Affiliations:** 1 School of Biosciences, University of Sheffield, Sheffield, UK; 2 School of Medicine and Population Health, University of Sheffield, Sheffield, UK

**Keywords:** *Enterococcus faecalis*, EPA, innate immune evasion, lipoproteins, phagocytosis

## Abstract

*Enterococcus faecalis* is an opportunistic pathogen frequently causing nosocomial infections. The virulence of this organism is underpinned by its capacity to evade phagocytosis, allowing dissemination in the host. Immune evasion requires a surface polysaccharide produced by all enterococci, known as the enterococcal poly-saccharide antigen (EPA). EPA consists of a cell wall-anchored rhamnose backbone substituted by strain-specific polysaccharides called ‘decorations’, essential for the biological activity of this polymer. However, the structural determinants required for innate immune evasion remain unknown, partly due to a lack of suitable validated assays. Here, we describe a quantitative, in vitro assay to investigate how EPA decorations alter phagocytosis. Using the *E. faecalis* model strain OG1RF, we demonstrate that a mutant with a deletion of the locus encoding EPA decorations can be used as a platform strain to express heterologous decorations, thereby providing an experimental system to investigate the inhibition of phagocytosis by strain-specific decorations. We show that the aggregation of cells lacking decorations is increasing phagocytosis and that this process does not involve the recognition of lipoproteins by macrophages. Collectively, our work provides novel insights into innate immune evasion by enterococci and paves the way for further studies to explore the structure/function relationship of EPA decorations.

## 1 Introduction

*Enterococcus faecalis* is a commensal bacterium found in the human digestive tract that can cause hospital- and community-acquired infections. In elderly patients, immunocompromised hosts or following antibiotic-induced dysbiosis, *E. faecalis* is often responsible for a wide variety of diseases including infective endocarditis and peritonitis, as well as infections at urinary catheter, and other surgical, sites (Arias & Murray, 2012). *E. faecalis* displays a high resistance to extracellular stressors including mild disinfectants (Rince et al., 2000) and antibiotics commonly used to treat bacterial infections such as cephalosporins (Djoric et al., 2020). The formation of biofilms is also a common feature of *E. faecalis*, further reducing the effectiveness of antibiotic treatments (Holmberg & Rasmussen, 2016). Multi-species biofilms are of particular concern since *E. faecalis* can augment the virulence of other bacteria (Montravers et al., 1997) and serve as a reservoir for antimicrobial resistance genes, particularly resistance to last-resort antibiotics such as vancomycin (Cong et al., 2020).

*E. faecalis* produces several virulence factors that have been studied in detail, but the exact mechanism of how this bacterium causes infections remains poorly understood. Virulence factors are not exclusively found in clinical isolates, and disease-causing strains can also colonise healthy individuals (Guzman Prieto et al., 2016). The use of zebrafish as an experimental model of infection revealed that the ability of *E. faecalis* to avoid uptake by innate immune cells (macrophages and neutrophils) is critical for pathogenesis (Prajsnar et al., 2013).

*E. faecalis* cell envelope composition and dynamics play an important role in resistance against innate immune effectors. Approximately, 40% of *E. faecalis* clinical isolates produce a capsular polysaccharide (Saffari et al., 2018), which masks opsonic C3 molecules from recognition by phagocytes (Thurlow et al., 2009). Meanwhile, there is evidence that non-opsonic phagocytosis is inhibited by enterococcal glycolipids (Diederich et al., 2014; Theilacker et al., 2011). The efficiency of *E. faecalis* uptake is further reduced by the activity of the autolysin AtlA, which prevents the formation of long chains of enterococci which are more readily phagocytosed (Salamaga et al., 2017). *E. faecalis* has also evolved mechanisms to survive innate immune effectors. Expression of aggregation substance, an envelope-localised adhesin, for example, facilitates entry into neutrophils (Vanek et al., 1999) and increases intracellular survival (Rakita et al., 1999).

The enterococcal polysaccharide antigen (EPA) is a cell envelope polymer produced by all enterococci that contributes to virulence (Palmer et al., 2012). EPA consists of a well-conserved rhamnose backbone decorated with covalently bound strain-specific polysaccharides called ‘decorations’ (Guerardel et al., 2020; Mistou et al., 2016). The chromosomal *epa* locus is subdivided into a conserved and a variable (*epa_var*) region. These two loci encode the biosynthetic machineries for the rhamnose backbone and the decoration polymers, respectively (Guerardel et al., 2020). Deletion of genes within either region significantly attenuates virulence (Prajsnar et al., 2013; Smith et al., 2019). Current research suggests that EPA helps to maintain cell envelope integrity, thus increasing resistance to antimicrobial peptides (Smith et al., 2019) and favouring intracellular survival (Da Silva et al., 2022). In addition, mutants lacking EPA decorations are avirulent in zebrafish and more susceptible to uptake by macrophages in vivo (Smith et al., 2019). The mechanisms by which EPA decorations inhibit phagocytosis remain unknown.

In this work, we describe a quantitative in vitro phagocytosis assay to investigate how *E. faecalis* cell surface components modulate phagocytosis. We provide the proof of concept that *E. faecalis* OG1RF with a complete deletion of the decoration locus can be used as a platform strain to investigate (i) the structure/function relationship of EPA by performing heterologous expression of strain-specific EPA decorations, and (ii) the recognition of cell envelope components by phagocytes in the absence of EPA decorations. Finally, we show that EPA decorations reduce phagocytosis by inhibiting the aggregation of enterococcal cells, thereby promoting dissemination in the host.

## 2 Results

### 2.1 Setting up an in vitro phagocytosis assay using IBMDMs

We sought to design an in vitro assay to quantitatively assess non-opsonic phagocytosis of *E. faecalis* without compounding effects from other immune processes. Immortalised bone marrow-derived macrophages (iBMDMs) from mice were utilised as model host phagocytes to measure the uptake of *E. faecalis* OG1RF derivatives constitutively expressing GFP (Salamaga et al., 2017). Following internalisation by iBMDMs, the number of intracellular bacteria was determined by proxy, measuring the green fluorescence intensity of individual iBMDM cells (Figure S1) (Boero et al., 2021).

**Figure 1:**
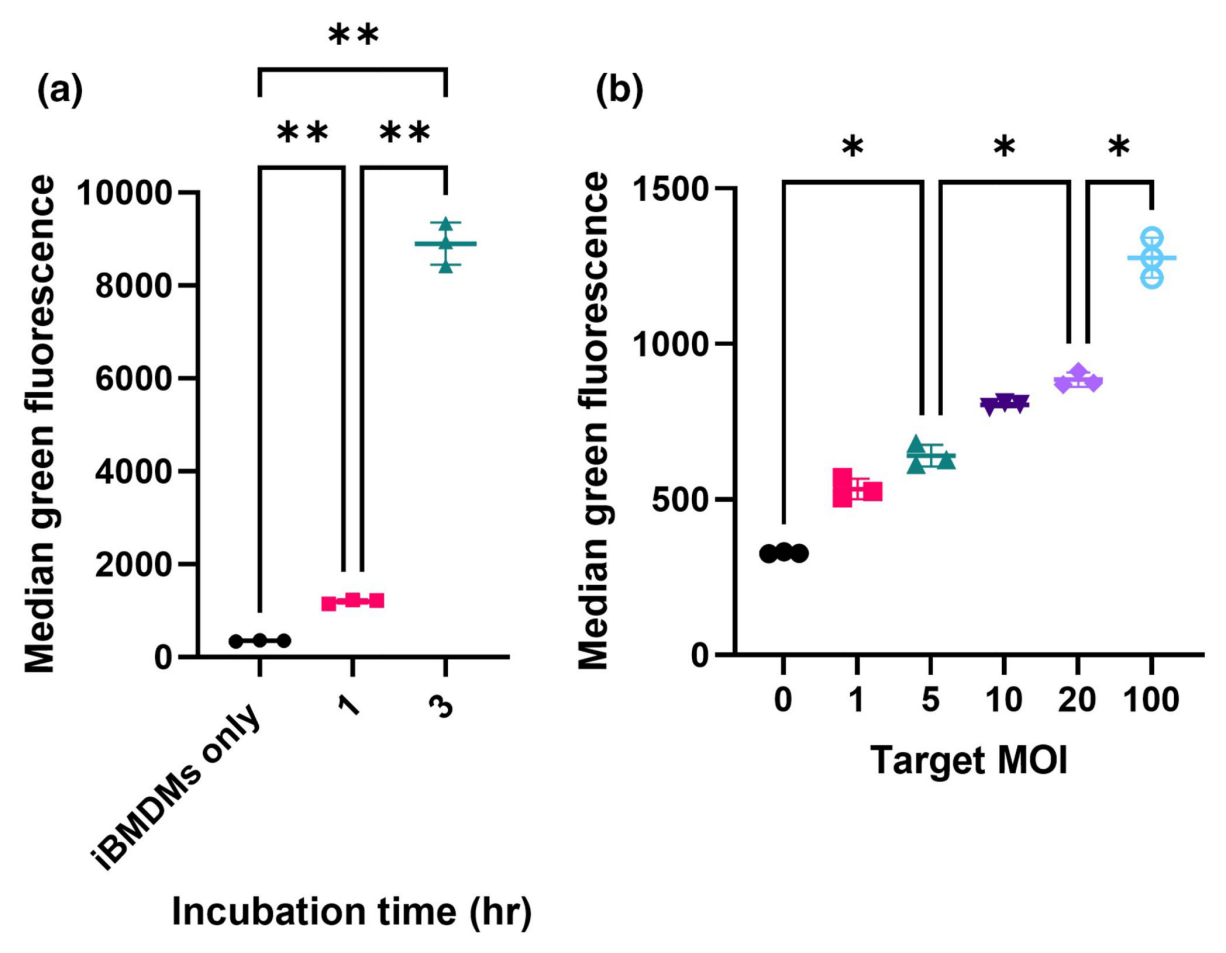
Setting up an assay to measure internalisation of GFP-labelled *E. faecalis* bacteria by iBMDMs. (a) Internalisation of *E. faecalis* after either 1 h or 3 h of incubation at 37°C. The graph shows the average brightness of macrophages that contained bacteria. To assess significance, a one-way ANOVA was performed, followed by Dunnett’s multiple comparisons test. *p*-values: IBMDMs only versus 1 h, **, *p* = 0.0023; iBMDMs only versus 3 h, **, *p* = 0.002; 1 h versus 3 h, **, *p* = 0.0025. (b) Internalisation of bacteria increases with increasing bacterial dose. Again, statistical analysis was performed via a one-way ANOVA followed by Dunnett’s multiple comparisons test. *p*-values: MOI = 0 versus MOI = 5, *, *p* = 0.0188; MOI = 5 versus MOI = 20, *, *p* = 0.0132; MOI = 20 versus MOI = 100, *, *p* = 0.0137. For (a) and (b), *N* = 3 technical replicates per condition, and error bars show mean values ± standard deviation (SD). *p*-value descriptors: *p < 0.05; **p < 0.01.

Before we compared the uptake of different strains, two critical conditions were optimised: incubation time and multiplicity of infection (MOI). First, OG1RF wild-type bacteria were incubated alongside iBMDMs for 1 h or 3 h at 37°C. Both test groups of iB-MDMs showed a significant increase in fluorescence as compared to the no-bacteria control, indicating that iBMDMs were internalising bacteria (Figure 1a). Fluorescence intensity associated with iBMDMs was much lower after a 1 h incubation as compared to after 3 hours. Based on these results, a 1 h incubation time was chosen for all future experiments, to enable the characterisation of mutants more readily uptaken. Next, wild-type bacteria were added to iBMDMs at an MOI of 0, 1, 5, 20 or 100 before co-incubation (1 h, 37°C). A dose–response was observed, in which iBMDM fluorescence increased with increasing MOI (Figure 1b). An MOI of 5 was chosen for future experiments, again to allow for the identification of mutants which are more readily phagocytosed.

Another way of quantifying phagocytosis was to determine the percentage of macrophages that had taken up bacteria. When looking at this metric over increasing time/MOI, the same trends were observed (Figures S2a–d and S3a–g), supporting the previous conclusions and showing that it was not just a subpopulation of iBMDMs internalising bacteria. Finally, it was demonstrated that uptake was significantly higher at 37°C compared to 4°C (Figure S4a,b), indicating that the fluorescence associated with iBMDMs results from active uptake of bacteria by phagocytosis (37°C) rather than bacteria binding to macrophage receptors (4°C controls) (Salman et al., 2000).

### 2.2 In vitro uptake by iBMDMs to explore EPA structure/function

**Figure 2:**
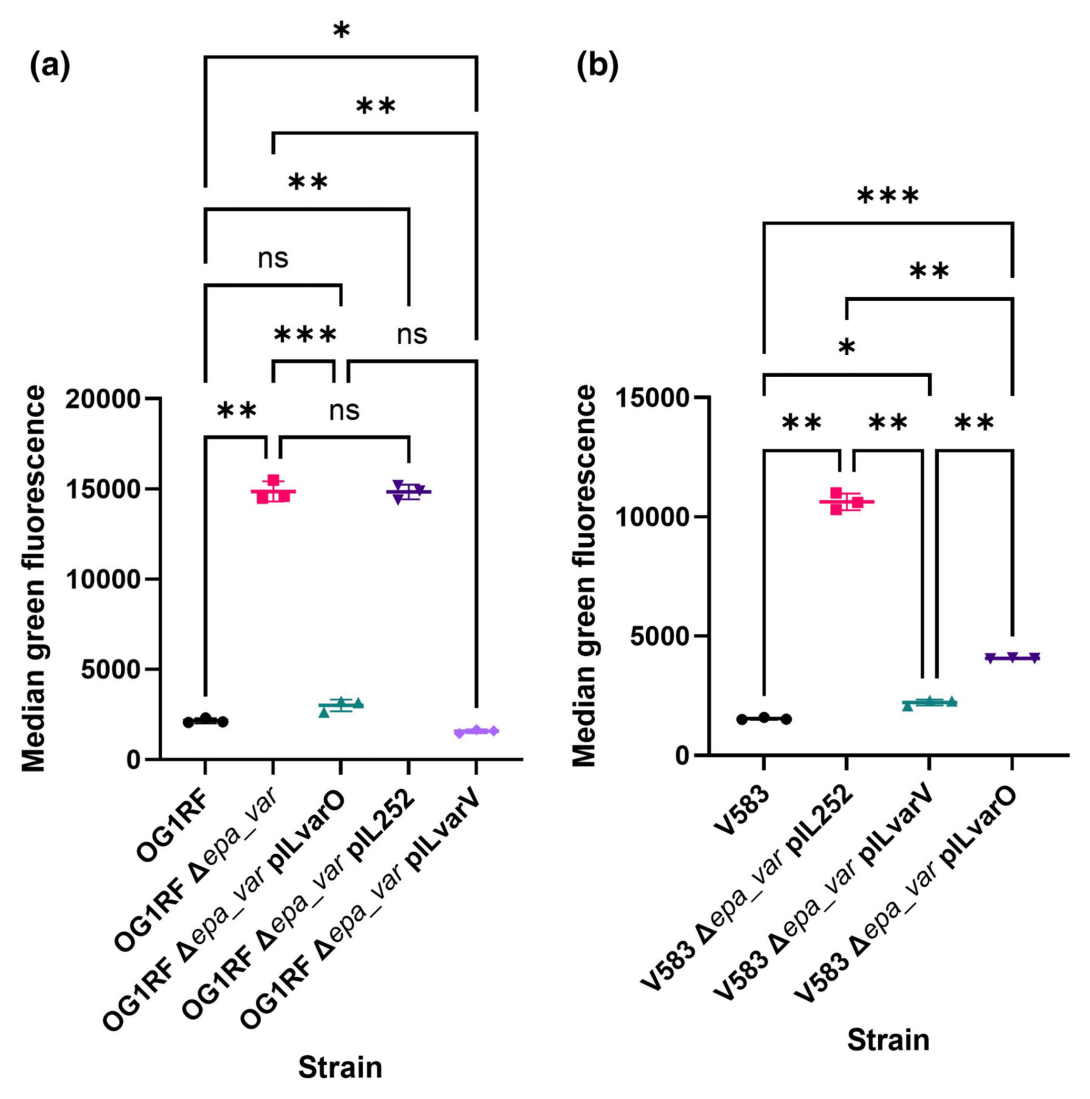
EPA decorations from strain V583 protect OG1RF Δ*epa_var* from phagocytosis, and vice versa. (a) Phagocytosis of OG1RF Δ*epa_var* transformed with an empty vector (pIL252) or a vector expressing V583 EPA decorations (pILvarV). Results are shown for one experiment with three technical replicates per group; these results are representative of three independent experiments. Statistical analysis was performed via one-way ANOVA with Brown–Forsythe and Welch’s correction followed by Dunnett’s multiple comparisons test. *p*-values: OG1RF versus Δ*epa_var*, **, *p* = 0.0025; OG1RF versus pILvarO, ns, *p* = 0.118; OG1RF versus pIL252, **, *p* = 0.0014; OG1RF versus pILvarV, *, *p* = 0.0274; Δ*epa_var* versus pILvarO, ***, *p* = 0.0003; Δ*epa_var* versus pIL252, ns, *p* > 0.999; Δ*epa_var* versus pILvarV, **, *p* = 0.0023; pILvarO versus pILvarV, ns, *p* = 0.0691. (b) Phagocytosis of V583 Δ*epa_var* transformed with pIL252 or a vector expressing OG1RF EPA decorations (pILvarO). Results are shown for one experiment with three technical replicates per group; these results are representative of three independent experiments. Statistical analysis was performed by via one-way ANOVA with Brown–Forsythe and Welch’s correction followed by Dunnett’s multiple comparisons test. *p*-values: V583 versus pIL252, **, *p* = 0.0015; V583 versus pILvarV, *, *p* = 0.0115; V583 versus pILvarO, ***, *p* = 0.0004; pIL252 versus pILvarV, **, *p* = 0.0020; pIL252 versus pILvarO, **, *p* = 0.0029; pILvarV versus pILvarO, **, *p* = 0.0047.

After optimising the conditions, the in vitro uptake assay was benchmarked using a mutant producing an EPA polysaccharide devoid of decorations (with a 17.6 kbp deletion of the *epa_var* region; strain Δ*epa_var*). As expected, the mutant displayed a significant increase in internalisation as compared to wild-type (Figure 2a, Figure S5a–e), confirming that EPA decorations facilitate escape from phagocytosis by macrophages. The mutant’s phenotype was fully complemented by a plasmid encoding the *epa_var* locus (Figure 2a; pILvar_O). The empty vector pIL252 had no significant impact on phagocytosis, confirming that protection was due to OG1RF decorations. Interestingly, there was no difference in the percentage of iBMDMs harbouring bacteria between wild-type, Δ*epa_var*, and complemented strains (Figures S5f and S6a,), but fluorescence intensity associated with iBMDMs significantly increased when mutant bacteria were administered (Figure S6b). Our findings were verified by performing fluorescence microscopy analysis on iBMDMs incubated with wild-type, mutant or complemented bacteria (Figure S6c,d).

With the assay benchmarked, we sought to investigate if we could compare the function of strain-specific EPA decorations by doing cross-complementation experiments (Furlan et al., 2019). As a proof of concept, we complemented the OG1RF Δ*epa_var* strain with a plasmid encoding the decoration from *E. faecalis* V583. Heterologous complementation revealed that V583 decorations offer a similar level of protection as OG1RF decorations (Figure 2a, Figure S5). This was also observed in the inverse experiment (Figure 2b, Figure S7a–e), where heterologous expression of OG1RF decorations significantly reduced the uptake of V583 Δ*epa_var* (as compared to the empty vector). Altogether, these findings show that strain-specific EPA decorations can cross-complement one another, suggesting a conserved protective mechanism.

### 2.3 Aggregation of the Δ*epa_var* mutant contributes to increased phagocytosis

The increase in median green fluorescence without an increase in the percentage of GFP-positive macrophages (Figure S6) suggested that more Δ*epa_var* cells are internalised as compared to wild-type cells. A defect in bacterial daughter cell separation, leading to the formation of longer bacterial cell chains, has been suggested to increase bacterial uptake by phagocytes (Salamaga et al., 2017). We therefore decided to investigate if the morphology of the *epa_var* mutant cells is contributing to an increased phagocytosis. We started by comparing growth of the wild-type, mutant, and complemented strains. When grown in BHI broth at 37°C, OG1RF Δ*epa_var* displayed a significant increase in doubling time compared to wild-type (Figure S8a,b), indicating that EPA decorations help to maintain normal bacterial growth.

**Figure 3:**
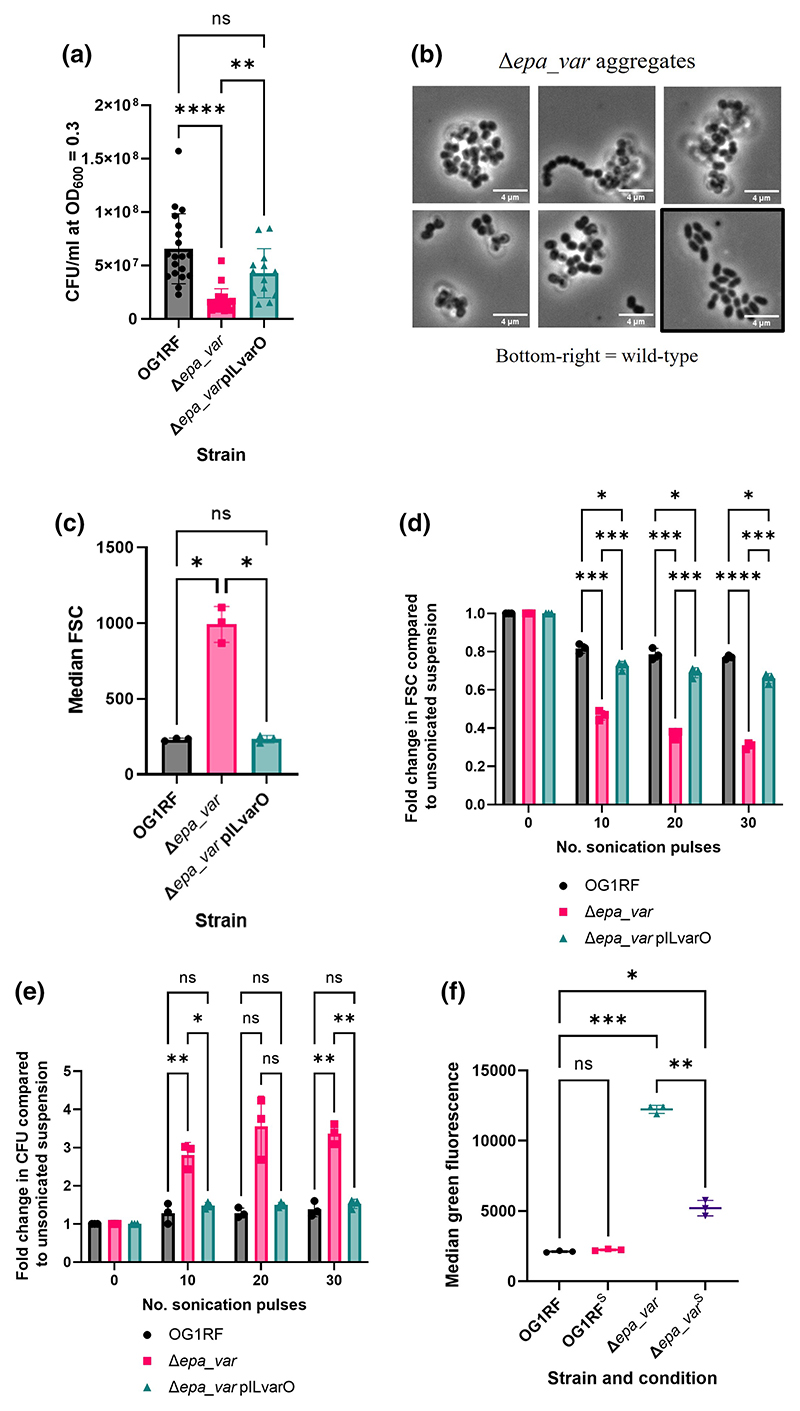
EPA decorations contribute to reduced internalisation by reducing bacterial cell aggregation. (a) CFU/mL of *E. faecalis* OG1RF Δ*epa_var* versus WT at OD_600_ = 0.3. Statistical analysis was performed by doing a one-way ANOVA with Brown–Forsythe and Welch’s correction, followed by Dunn’s multiple comparisons test. *p*-values: OG1RF versus Δ*epa_var*, ****, *p* < 0.0001; OG1RF versus pILvarO, ns, *p* = 0.0769; Δ*epa_var* versus pILvarO, **, *p* = 0.0062. Number of biological replicates per group: WT, *n* = 19; Δ*epa_var, n* = 20; Δ*epa_var* pILvarO, *n* = 13. (b) Representative phase contrast images of aggregates formed by *E. faecalis* OG1RF Δ*epa_var* bacteria. A representative image of WT bacteria (bottom right, boxed) is provided for comparison. (c) Median FSC of early exponential-phase OG1RF WT, Δ*epa_var* or Δ*epa_var* complemented bacteria. For each group, *n* = 3 biological replicates. Statistical analysis was performed via a one-way ANOVA with Brown–Forsythe and Welch’s correction, followed by Dunnett’s multiple comparisons test. *p*-values: OG1RF versus Δ*epa_var*, *, *p* = 0.0167; OG1RF versus pILvarO, ns, *p* = 0.996; Δ*epa_var* versus pILvarO, *, *p* = 0.0174. (d) Fold decrease in bacterial particle FSC compared to suspensions before sonication. Three biological replicates per group. Statistical analysis was performed by doing a two-way ANOVA followed by Tukey’s multiple comparisons test. No. pulses = 10: OG1RF versus Δ*epa_var*, ***, *p* = 0.0002; OG1RF versus pILvarO, *, *p* = 0.0227; Δ*epa_var* versus pILvarO, ***, *p* = 0.0004. No. pulses = 20: OG1RF versus Δ*epa_var*, ***, *p* = 0.0002; OG1RF versus pILvarO, *, *p* = 0.0320; Δ*epa_var* versus pILvarO, ***, *p* = 0.0002. No. pulses = 30: OG1RF versus Δ*epa_var*, ****, *p* < 0.0001; OG1RF versus pILvarO, *, *p* = 0.0211; Δ*epa_var* versus pILvarO, ***, *p* = 0.0004. (e) Fold increase in CFU count compared to bacterial suspensions before sonication. All counts were normalised to OD_600_ = 0.3. Three biological replicates per group. Statistical analysis was performed by doing a two-way ANOVA followed by Tukey’s multiple comparisons test. No. pulses = 10: OG1RF versus Δ*epa_var*, **, *p* = 0.0082; OG1RF versus pILvarO, ns, *p* = 0.537; Δ*epa_var* versus pILvarO, *, *p* = 0.0278. No. pulses = 20: OG1RF versus Δ*epa_var*, ns, *p* = 0.0651; OG1RF versus pILvarO, ns, *p* = 0.162; Δ*epa_ var* versus pILvarO, ns, *p* = 0.0821. No. pulses = 30: OG1RF versus Δ*epa_var*, **, *p* = 0.0015; OG1RF versus pILvarO, ns, *p* = 0.580; Δ*epa_var* versus pILvarO, **, *p* = 0.0043. (f) iBMDM-mediated phagocytosis of sonicated (^S^) or unsonicated bacteria. Sonicator settings = 20 pulses using 20% amplitude. In this experiment, three technical replicates were performed per group. Statistical analysis was performed using a one-way ANOVA with Brown–Forsythe and Welch’s correction followed by Dunnett’s multiple comparisons test. *p*-values: OG1RF versus OG1RF^S^, ns, *p* = 0.247; OG1RF versus Δ*epa_var*, ***, *p* = 0.0009; OG1RF versus Δ*epa_var*^S^, *,; z*p* = 0.0317; Δ*epa_var* versus Δ*epa_var*^S^, **, *p* = 0.0011. Key to *p*-values: ns, not significant; *, *p* < 0.05; **, *p* < 0.01; ***, *p* < 0.001; ****, *p* < 0.0001.

We noticed that the Δ*epa_var* mutant consistently showed fewer CFU counts versus wild-type cells when plated. To investigate this formally, serial dilutions of exponential cultures were plated and CFU counts were made and normalised to OD_600_ = 0.3. Δ*epa_var* exponential cultures consistently showed a decrease in CFU/mL compared to both the wild-type and the complemented strain (Figure 3a). To investigate the phenotypes observed in more detail, exponential-phase Δ*epa_var* bacteria were analysed via confocal microscopy. Peptidoglycan cell wall shape and septum formation were visualised by labelling the bacteria with an Alexa555 NHS ester and a fluorescent D-amino acid (HADA), respectively (Figure S9a). Mutant bacteria displayed single septa running perpendicular to the direction of cell division, suggesting that division was occurring normally. However, when compared to wild-type and complemented bacteria, Δ*epa_var* bacterial cells were significantly shorter in length and greater in width, giving them a more spherical appearance (Figure S9b–d). In addition, microscopic analysis revealed more evidence that Δ*epa_var* bacteria form aggregates, with large, amorphous clumps of bacteria prevalent (Figure 3b). In contrast, wild-type bacteria tended to be arranged as more discrete diplococci. To quantify the putative aggregation phenotype, FSC measurements were taken for wild-type, mutant, and complemented bacteria via flow cytometry (Figure 3c). Δ*epa_var* bacteria displayed a significant increase in FSC, which is consistent with the formation of aggregates. Sonication of Δ*epa_var* bacteria decreased FSC and increased CFU count, which is also consistent with the bacterial cell aggregation hypothesis (Figure 3d,e). The separation of bacterial aggregates in the *epa_var* mutant partially restored the uptake by iBMDMs, indicating that the formation of aggregates contributes to increased uptake (Figure 3f, Figure S10a–e).

### 2.4 Recognition of surface lipoproteins is not responsible for the increased phagocytosis in the absence of EPA decorations

**Figure 4:**
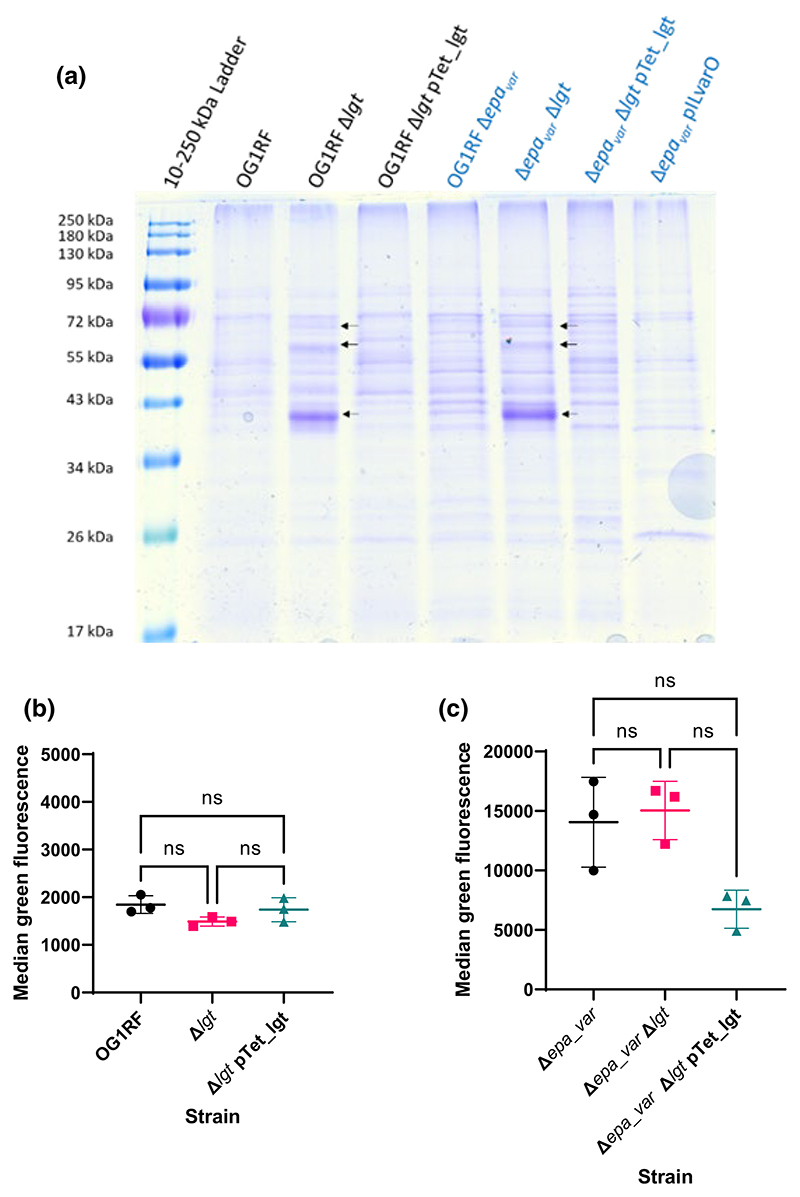
Detection and characterisation of OG1RF Δ*lgt* and Δ*epa_var* Δ*lgt* mutants. (a) Proteins released into the culture supernatant by *E. faecalis* cells at exponential phase (OD600 = 0.3). Deletion of *lgt* results in additional proteins shed (black arrows). (b) Phagocytosis of OG1RF Δ*lgt*. Statistical analysis was performed via one-way ANOVA with Brown–Forsythe and Welch’s corrections, followed by Dunnett’s multiple comparisons test. *p*-values: OG1RF versus Δ*lgt, p* = 0.317; OG1RF versus pTet_lgt, *p* = 0.999; Δ*lgt* versus pTet_lgt, *p* = 0.771. Sample sizes: *n* = 3 biological replicates per group. (c) Phagocytosis of Δ*epa_var* Δ*lgt*. Same statistical analysis method as (b). *p*-values: OG1RF versus Δ*lgt, ns, p* > 0.999; OG1RF versus pTet_lgt, ns, *p* = 0.286; Δ*lgt* versus pTet_lgt, ns, *p* = 0.0951. Sample sizes: *n* = 3 biological replicates per group. Key to *p*-values: ns, not significant.

The presence of EPA decorations at the cell surface prevents other cell envelope components from being recognised by immune receptors. In group B streptococci, the capsular polysaccharide masks a streptococcal lipoprotein from being recognised by macrophages by scavenger receptor A (Areschoug et al., 2008). Enterococcal lipoproteins are known to activate pro-inflammatory signalling cascades (Zou & Shankar, 2016) and may contribute to *E. faecalis*-associated intestinal inflammation (Ocvirk et al., 2015). To test the role of lipoproteins in uptake, we generated *E. faecalis* OG1RF mutants with an in-frame deletion of *lgt* (*OG1RF_11459*) in both the wild-type (Figure S11a) and Δ*epa_var* backgrounds (Figure S11b). This gene encodes prolipoprotein diacylglyceryl transferase, the enzyme responsible for anchoring lipoproteins onto the enterococcal cell surface (Buddelmeijer, 2015). A deletion of *lgt* in *E. faecalis* V583 led to an increase in lipoprotein shedding into the culture supernatant (Reffuveille et al., 2012). We used a TCA-based precipitation method to purify proteins from culture supernatants and profile them via SDS-PAGE (Figure 4a). More protein species were indeed detected in Δ*lgt* culture supernatants than were seen in parental ones. Furthermore, this phenotype could be complemented with an inducible expression system. Altogether, these findings suggest that our mutants lack Lgt activity.

Next, we measured the uptake of these mutants by iMBDMs (Figure 4b,c). Deletion of *lgt* did not lead to any significant change in phagocytosis, irrespective of the production of EPA decorations. This suggests that immune evasion is not due to EPA decorations masking lipoproteins.

## 3 Discussion

EPA decorations facilitate *E. faecalis* virulence by mediating resistance to extracellular stressors and phagocytosis (Smith et al., 2019). We established an in vitro phagocytosis assay using iBMDMs and determined optimum conditions to detect uptake for both wild-type and mutants with an altered cell envelope.

We established that V583 EPA decorations complement OG1RF Δ*epa_var* and vice versa, strongly suggesting that EPA decorations facilitate immune evasion via a conserved mechanism (Figure 2a,b). The genetic loci encoding EPA decorations in strains OG1RF and V583 are strikingly different. Yet, the expression of both loci in the OG1RF Δ*epa_var* background can inhibit phagocytosis. Structural studies are required to establish if both decorations share motifs sufficient to protect against uptake by macrophages. However, it is tempting to assume that the architecture of EPA, irrespective of its composition, is masking enterococcal cell envelope components from being bound by phagocytic receptors. In future, it would be interesting to expand our EPA cross-complementation study to cover a greater structural diversity of decorations. From this proposed work, it may be possible to define the EPA structural requirements critical for immune evasion or establish that EPA decoration structure is not important so long as a protective barrier is formed.

Our results suggest that the presence of EPA decorations at the cell surface is required to limit bacterial aggregation and thereby minimises the number of bacteria taken up by phagocytes. Given that EPA decorations have a net negative charge (Smith et al., 2019), we postulate that the decorations reduce aggregation by inhibiting hydrophobic interactions between bacteria. Measurements performed independently by different research groups have consistently shown that the deletion of *epa* genes increases enterococcal surface charge and hydrophobicity (Korir et al., 2019; Rouchon et al., 2022; Smith et al., 2019). The cell aggregates formed by Δ*epa_ var* are more efficiently internalised by macrophages. This conclusion is supported by the fact that GFP-expressing Δ*epa_var* bacteria increase macrophage fluorescence without increasing the percentage of macrophages positive for bacteria (Figure S4). Dispersion of bacterial aggregates by sonication significantly reduces internalisation. This result is consistent with a previous study showing that the minimisation of bacterial cell size is an important factor for the dissemination of *E. faecalis* in the host (Salamaga et al., 2017). A similar mechanism has been reported for *Streptococcus pneumoniae*, which also minimises phagocytic uptake by minimising aggregation (Dalia & Weiser, 2011). In contrast, uropathogenic *E. coli* seem to inhibit phagocytosis by morphing into long, filamentous cells whose elongated shape makes phagocytic cup formation less mechanistically favourable (Justice et al., 2004; Möller et al., 2012). This illustrates the diversity of mechanisms evolved by bacteria to circumvent phagocytosis.

In the absence of EPA, the enterococcal cell surface can be readily recognised by iBMDMs. Our study indicates that the PAMP(s) responsible for this recognition are not membrane-anchored lipoproteins and therefore remain to be identified. These could be cell wall-anchored proteins, peptidoglycan, rhamnan or lipoteichoic acids (LTAs). Testing the contribution of some of these components to phagocytic uptake will be challenging. In the presence of LTA synthase (LtaS) inhibitors, *Enterococcus faecium* cells displayed severe growth and morphological defects (Paganelli et al., 2017), suggesting that LTAs are essential for this genus. Attempts to delete both enterococcal homologues of LtaS in *E. faecalis* were unsuccessful (data not shown), further suggesting that LTAs are essential in enterococci. A different approach to modulate the abundance of LTAs may represent an alternative strategy to test.

Recent work has shown that iBMDMs represent a genetically tractable system (Covarrubias et al., 2017). This cell line can easily be transfected, and a CRISPR/Cas9 gene deletion system has been successfully used. Access to these tools will therefore enable further studies to explore specific TLR receptors involved in *E. faecalis* recognition and signalling pathways.

The scope of this study was limited to non-opsonic phagocytosis. It has been shown elsewhere that an *E. faecalis* V583 EPA decoration mutant is more readily bound by two complement components—mannose-binding lectin and C3b—leading to increased neutrophil-mediated opsonophagocytosis (Geiss-Liebisch et al., 2012). Therefore, it would be interesting to investigate the mechanisms (if any) by which EPA decorations in other strains inhibit this process.

**Figure 5:**
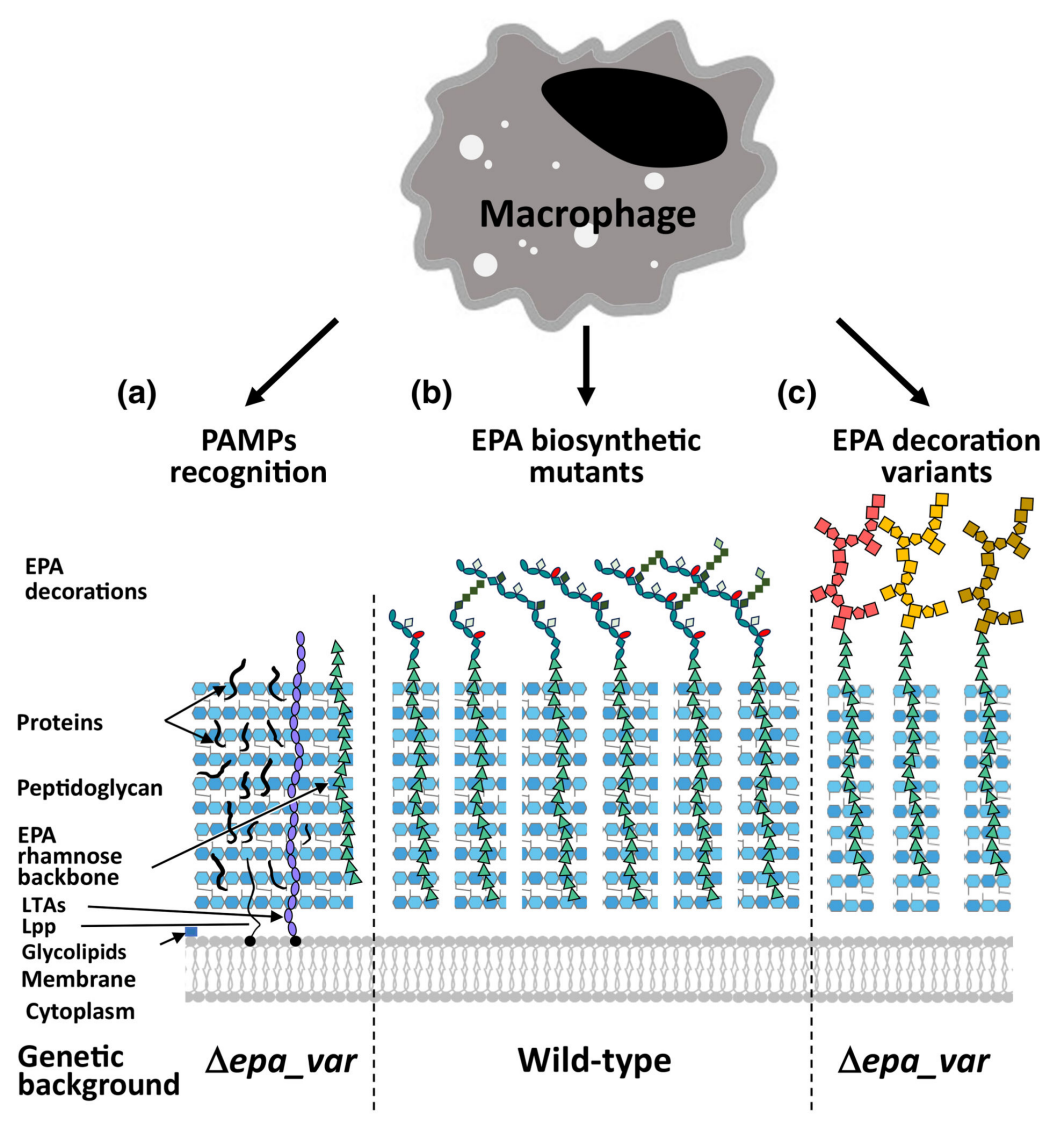
A phagocytosis assay to explore *E. faecalis* interaction with innate immune cells. The assay described in this study can be used to identify the PAMPs recognised by phagocytes (a), looking for a decreased uptake of mutants built in the OG1RF Δ*epa_var* background. The analysis of EPA structure/function using NMR and the phagocytosis assay (b) will provide insights into the biosynthesis of decorations and the specific contribution of structural determinants to innate immune evasion. The OG1RF Δ*epa_var* can also be used to test the biological activity of EPA decorations produced by *E. faecalis* isolates (c). LTAs, lipoteichoic acids; Lpp, lipoproteins.

The assay described in this study represents a tool to explore the contribution of cell envelope components to innate immune evasion and recognition by phagocytes (Figure 5). This versatile assay can be used for several purposes: (i) to identify the Pathogen-Associated Molecular Patterns (PAMPs) recognised by phagocytes (Figure 5a), looking for a decreased adhesion/uptake and killing of mutants built in the OG1RF Δ*epa_var* background; (ii) to explore EPA decorations structure/function (Figure 5b); and (iii) to test the biological activity of EPA decorations produced by *E. faecalis* isolates (Figure 5c).

## 4 Experimental Procedures

### 4.1 Bacterial strains and growth conditions

All bacterial strains used in this work are listed in Table S1. Unless stated otherwise, *E. faecalis* was cultured by inoculating a single colony into Brain Heart Infusion (BHI) broth and incubating at 37°C without agitation. *E. faecalis* colonies were cultivated on 1.5% (w/v) BHI agar plates at 37°C; these plates were stored at 4°C for up to 1 month. When appropriate, media/agar was supplemented with antibiotics to maintain selection of plasmids (Table S1). To promote the expression of genes on pTetH2op derivatives, anhydrotetracycline (ATc) was added to a final concentration of 10 ng μL^−1^. *E. coli* work was performed as follows: Unless stated otherwise, a single colony was inoculated into BHI or Luria-Bertani (LB) broth for incubation at 37°C with agitation. Single colonies were cultivated on 1.5% (w/v) BHI agar plates at 37°C. When appropriate, antibiotics were added as described in (Table S2).

### 4.2 Construction of GFP-expressing *E. faecalis*

The plasmid pMV_GFP was electroporated into *E. faecalis* electrocompetent cells. Transformants were selected on BHI agar +5 μg/mL tetracycline plates at 37°C. GFP expression was verified by imaging patched transformants on a Gel DocTM XR+ imager (Alexa488 channel).

### 4.3 Tissue culture

Immortalised bone marrow-derived macrophages (iBMDMs) from wild-type mice were isolated using standard procedures (Hornung et al., 2008). They were obtained from the BEI Resources, NIAID NIH (NR-9456) (https://www.beiresources.org/Catalog/cellBanks/NR-9456.aspx). The characterisation of iBMDMs based on immunofluorescence, stimulation assays and flow cytometry demonstrated that it retains its macrophage-specific morphological, functional and surface expression properties (Hornung et al., 2008). iBMDMs were cultured in DMEM (Gibco) supplemented with 1% (v/v) foetal bovine serum (FBS, PAN Biotech; low endotoxin, heat inactivated), penicillin (10 U/mL)/streptomycin (1 mg/mL) (Lonza) and 1% (v/v) sodium pyruvate (Thermo Fisher, 1 mM final concentration). Cells were cultured in standard tissue culture flasks or multi-well plates at 37°C in 5% CO_2_, washed in PBS and given fresh media once every 48 h. Cells were split when >70% confluence had been reached.

### 4.4 In vitro internalisation assay optimisation

One hour versus three hours experiment: on Day 1, iBMDMs were checked for >70% confluence. An exact cell count was made using a Countess automated cell counter (Invitrogen) as per the manufacturer’s instructions. iBMDMs were diluted to 4 × 10^5^ live cells per mL in fresh media. To set up one technical replicate, a 5 mL (2 × 10^6^ cells) was transferred to a 25 mL tissue culture flask. Flasks were incubated as normal. In addition, one *E. faecalis* OG1RF pMV_GFP overnight culture was set up in 10 mL BHI + 5 μg/mL tetracycline. On Day 2, iBMDMs were washed once with PBS and given 1 mL fresh DMEM (serum- and antibiotic-free). Bacteria were harvested (5 min at 4000 × *g*) and resuspended in PBS. Optical density of bacterial suspensions was normalised to OD_600_ = 1 (1 × 10^9^ CFU/mL). Bacterial suspension was 10× diluted in DMEM (serum- and antibiotic-free), giving 1 × 10^8^ CFU/mL. One mL of this suspension was added to each iBMDM flask (MOI = 50), and then flasks were incubated for 1 h or 3 h at 37°C, 5% CO_2_. For the 3 h on ice control, flasks were incubated on ice for 5 min prior to addition of bacteria. Post-incubation, iBMDMs were washed three times with PBS and treated with 5 mL DMEM +250 μg/mL gentamycin +20 μg/mL vancomycin for 1 h at 37°C, 5% CO_2_. Then, iBM-DMs were washed twice with PBS, resuspended in 5 mL PBS using a cell scraper, and transferred to 15 mL Falcon tubes. iBMDMs were pelleted (5 min at 4000 × *g*) and resuspended in 1 mL PBS + 4% (m/v) paraformaldehyde for 10 min fixing at room temperature. iBMDMs were re-pelleted, washed once with PBS, resuspended in 500 μL filtered-sterilised PBS and stored at 4°C in darkness until Day 3.

MOI dose–response experiment: the method used was mostly the same as described above, but with the changes outlined here: On Day 1, confluent iBMDMs were diluted to 1 × 10^5^ live cells/mL. Two mL (2 × 10^5^ iBMDMs) were transferred to each well on a six-well plate. On Day 2, an *E. faecalis* suspension was prepared as above, and increasing volumes were added to iBMDMs to give MOI = 1, 5, 10, 20 or 100. The volume of DMEM added (serum- and antibiotic-free) added to each well was adjusted so total volume = 2 mL. Incubation time = 1 h (37°C, 5% CO_2_). In total, three six-well plates were used to perform three technical replicates per MOI plus bacteria-free control wells. After incubation, the method used was the same as described above, except that (i) the volume of DMEM + gentamycin + vancomycin was 2 mL per well, (ii) the volume of PBS used for washing/resuspending was 1 mL, and (iii) the volume of 4% PFA used for fixing was 500 μL.

### 4.5 In vitro internalisation assays to compare uptake of different *E. faecalis* strains

On Day 1, iBMDMs were checked for >70% confluence and counted. iBMDMs were diluted to 2.5 × 10^5^ live cells per mL in fresh media; 5 × 10^5^ cells were aliquoted per well. *E. faecalis* overnight cultures were set up as standard. On Day 2, fresh *E. faecalis* cultures were started (100 μL overnight into 10 mL fresh media) and grown at 37°C until OD_600_ = 0.3. Cultures were pelleted (5 min at 4000 × *g*) and resuspended in an equal volume of DMEM (serum- and antibiotic-free). iBMDMs, after being washed and given fresh media as before, were given 2.5 × 10^6^ CFU bacteria per well (MOI = 5). Three wells were allocated per bacterial strain in each experiment. After 1 h at 37°C in 5% CO_2_, iBMDMs were washed and treated with antibiotics as before. Then, cells were washed twice with PBS and detached by treating with 1 mL Accutase™ (Merck) for 30 min at 37°C in 5% CO_2_. Detached iBMDMs were pelleted (5 min at 7000 × *g*), fixed, resuspended in 200 μL filtered PBS and stored at 4°C in darkness.

### 4.6 Sonication of *E. faecalis* Δ*epa_var* cells

A 5 mL aliquot of each bacterial suspension was treated with 20 cycles of sonication (5 s at 20% amplitude) using a Fisherbrand™ 505 sonicator (Fisher).

### 4.7 Flow cytometry analysis of iBMDMs

200 μL iBMDM samples were vortexed gently and transferred to a 96-well plate. Data acquisition was performed using a Guava easy-Cyte HT flow cytometer (Luminex). Data analysis was carried out using guavaSoft version 3.1.1; gating strategy is shown in (Figure S1).

### 4.8 Fluorescence microscopy of iBMDMs

In vitro phagocytosis assay was performed exactly as above. 100 μL iBMDM samples were transferred to a 24-well plate. Each sample was diluted by adding 1 mL of PBS. iBMDMs images were captured with Elements software (Nikon) using an Andor Neo camera on Nikon Ti microscope with differential interference contrast (DIC) and GFP epifluorescence. In (Fiji is just) ImageJ version 2.9.0/1.5 t, iBMDMs that had overlapping GFP signals were identified, and the fluorescence was quantified as mean grey value (MGV). MGV measurements were normalised by subtracting the average MGV of the background of the image. For each group, >90 macrophages were measured.

### 4.9 *E. faecalis* growth curves

*E. faecalis* overnight cultures (three per strain) were set up as normal. The next morning, each overnight culture was serially diluted in a 96-well plate. Each dilution step meant transferring 20 μL culture to 180 μL fresh BHI broth (i.e. a 10-fold dilution). After the final dilution, each culture had been diluted by 1 × 10^3^. The plate’s lid was replaced only after it had been treated with a solution of 0.05% (v/v) Triton X-100 + 20% (v/v) ethanol to prevent condensation. The plate was loaded into a Sunrise™ microplate reader (Tecan), and growth was allowed to proceed for 24 h at 37°C. Optical density (OD) measurements were taken every 5 min (wavelength = 595 nm). Cultures were agitated for 5 s at normal power before each measurement. Once the run had been completed, each curve was plotted as OD_595_ (*y*-axis, logarithmic) versus time in minutes (*x*-axis, linear).

### 4.10 CFU/mL determination of exponential *E. faecalis* cultures

*E. faecalis* cultures were set up by using 100 μL overnight culture to inoculate 10 mL fresh BHI broth. Cultures were incubated at 37°C without agitation until OD_600_ = 0.3. Ten-fold serial dilutions were performed in PBS until the cultures had been diluted by 1 × 10^7^. 100 μL of each final dilution was plated onto a standard BHI agar plate and incubated overnight at 37°C. The next morning, each plate was placed under a Scan4000 automated colony counter (Interscience). By calculating backwards from the CFU counts, CFU/mL values of the undiluted cultures were determined and normalised to OD_600_ = 0.3. A mean CFU/mL value was determined for each strain from at least three independent cultures.

### 4.11 Fluorescence microscopy of *E. faecalis*

*E. faecalis* was grown until OD_600_ = 0.3. One mL of each culture was pelleted (6000 × *g*, 1 min), resuspended in 1 mL leftover culture, and stained with 5 μL of 50 mM HADA (10 min on a rotary shaker at 37°C in complete darkness). Bacteria (kept wrapped in foil to prevent photobleaching) were pelleted as before, washed twice with PBS and resuspended in 300 μL of PBS. Next, bacteria were supplemented with 5 μL of AlexaFluor™ 555 NHS ester (Molecular Probes) at a concentration of 1 mg/mL and left to be stained for 7 min at room temperature. As a fixing step, bacteria were pelleted, resuspended in 750 μL 4% (m/v) paraformaldehyde in PBS and left for 30 min at room temperature. After fixing, cells were washed twice in PBS and resuspended in 20 μL of MilliQ water. Five μl were mounted onto a PolyPrep slide using SlowFade™ Gold (Thermo Fisher) and a standard 13 mm coverslip. Images were captured on a Nikon DualCam system (Eclipse Ti inverted research microscope). Wavelengths and filters (Table S3) were applied as appropriate for each image. Contrast and brightness adjustments were made in ImageJ.

### 4.12 Phase contrast microscopy of *E. faecalis*

*E. faecalis* strains were grown to OD_600_ = 0.3; then a 1 mL aliquot of each culture was pelleted (6000 × *g*, 1 min). Bacteria were fixed in 750 μL 4% (m/v) paraformaldehyde as described in the previous section. Fixed bacteria were washed twice in PBS, resuspended in 20 μL of MilliQ water and mounted as described previously. Images were captured on a Nikon DualCam system (Elipse Ti inverted research microscope). Cell length and width measurements were made using the ObjectJ plugin in ImageJ.

### 4.13 Flow cytometry analysis of *E. faecalis*

*E. faecalis* strains were grown to OD_600_ = 0.3, pelleted (4000 × *g*, 5 min) and resuspended in PBS at an OD_600_ of 0.4. Bacterial suspensions were treated with 10, 20 and 30 pulses using the Fisherbrand™ 505 sonicator. After every 10 pulses, 200 μL of the cell suspension was taken out and serially diluted (10-fold dilutions until diluted by 1 × 10^4^). To measure CFU/mL, two 100 μL aliquots of each final dilution were plated and CFU were counted using the Scan4000 colony counter (Interscience). To measure the forward scatter (FSC), 10 × dilutions of bacterial suspensions were passed through the Guava easyCyte HT, followed by data processing and analysis as described in (Figure S12). To provide a negative control, we also analysed some bacterial suspension that was set aside and not sonicated.

### 4.14 Construction of pG_lgt for allelic replacement

Plasmids and oligos used in this study are listed in Table S1. Two homology regions flanking the *lgt* open reading frame were amplified from OG1RF genomic DNA via PCR. The 5′ arm (~0.75 kb) was amplified using the primers SM_0194 (sense) and SM_0195 (antisense), whereas the 3′ arm (~0.75 kb) was amplified using SM_0196 (sense) and SM_0197 (antisense). Once purified, the two PCR products were mixed (equimolar amount of each) and fused into a single product (~1.5 kb) via splice overlap extension PCR (Ho et al., 1989) using primers SM_0194 and SM_0197. The resulting fragment was cut by *Xho*I and *Not*I and cloned into pGhost9 vector cut with the same enzymes (Maguin et al., 1992). Candidate pGhost derivatives were screened by PCR using primers SM_0171 and SM_0172. A positive clone containing the fused H1-H2 insert was checked by sanger sequencing, and the corresponding plasmid was named pG_lgt.

### 4.15 Construction of *E. faecalis* Δ *lgt* mutants

*E. faecalis* mutants were built by allelic exchange as previously described (Mesnage et al., 2008). Purified pG_lgt plasmid was electroporated into *E. faecalis* OG1RF wild-type and OG1RF Δ*epa_var*. Transformants were selected on BHI agar +30 μg/mL erythromycin plates at 28°C (a plasmid replication-permissive temperature). Transformants were then streaked onto BHI agar 30 μg/mL erythromycin without antibiotic at 42°C (a non-replication-permissive temperature) to select plasmid single-crossover recombination events. Colonies from these plates were used to inoculate BHI broth cultures and passaged repeated at 28°C without antibiotic. To find double-crossover recombination events, single colonies were re-isolated and screened via PCR using the primers SM_0210 and SM_0211 (Table S1). Double crossovers – corresponding to Δ*lgt* mutants—were identified in both backgrounds and validated by purifying their genomic DNA sequencing the *lgt* locus.

### 4.16 Complementation of *E. faecalis* Δ *lgt* mutants

The complete *lgt* gene was PCR amplified using primers SM_0401 and SM_0402 and cloned into the pTetH vector using NcoI and BamHI. Candidates were screened by using primers SM_0100 and SM_0101. A positive clone containing the *lgt* insert was checked by sanger sequencing, and the corresponding plasmid was named pTetH_lgt. *lgt* expression was induced by adding 10 ng/mL anhydrotetracycline.

### 4.17 Preparation of protein extracts from *E. faecalis* culture supernatants

*E. faecalis* was grown to OD_600_ = 0.3 as normal. Proteins contained in 1.8 mL of culture were precipitated by adding 200 μL 100% (w/v) trichloroacetic acid (TCA). After 15 min on ice, the samples were spun (25,000 × *g*, 10 min at 4°C). Proteins were washed in μL acetone, centrifuged (25,000 × *g*, 5 min at 4°C) and left to dry. Pellets were resuspended in 95 μL PBS + 5 μL Tris base and stored at−80°C.

### 4.18 SDS-PAGE

SDS-PAGE was performed as previously described (Laemmli, 1970). Protein extracts were mixed with 5× loading dye (250 mM Tris–HCl (pH 6.8), 10% (w/v) SDS, 0.5% (w/v) bromophenol blue, 50% (v/v) glycerol, 0.5 M dithiothreitol). Gels were stained with a Coomassie solution (0.25% (w/v) Coomassie blue R-250, 50% (v/v) methanol, 10% (v/v) glacial acetic acid) for 1 h at room temperature with gentle rocking and destained in 5% (v/v) methanol and 10% (v/v) glacial acetic acid.

### 4.19 Statistical analysis

GraphPad Prism version 10.1.2 was utilised for statistical analysis. Unless stated otherwise, all error bars on graphs represent mean ± SD. Each set of iBMDM flow cytometry data was analysed using a one-way ANOVA with Welch’s correction, followed by Dunnett’s multiple comparisons test. *E. faecalis* doubling times, CFU/mL at early exponential phase, *E. faecalis* cell length/width, and *E. faecalis* FSC between strains were also analysed in this manner. Since there were only two groups in the 37°C versus 4°C phagocytosis assay, an unpaired, two-tailed *t*-test with Welch’s correction was used here. iBMDM microscopy data were analysed using a Kruskal–Wallis test followed by Dunn’s multiple comparisons test. For the experiment comparing *E. faecalis* strain versus FSC versus number of pulses, a two-way ANOVA was performed, followed by Tukey’s multiple comparisons test.

## Supplementary Material

Additional supporting information can be found online in the Supporting Information section at the end of this article.

Supplementary Material

## Data Availability

Raw data and materials described in this study are available upon request.
