# Supplementary material for: Exploring the role of E. faecalis enterococcal polysaccharide antigen (EPA) and lipoproteins in evasion of phagocytosis
Source: Mol Microbiol. Author manuscript; Available in PMC 2025 Sep 9. (PMC7618083; doi:10.1111/mmi.15294)
Supplement: Supplementary Material [file EMS208353-supplement-Supplementary_Material.zip › mmi15294-sup-0001-figures1.pdf]

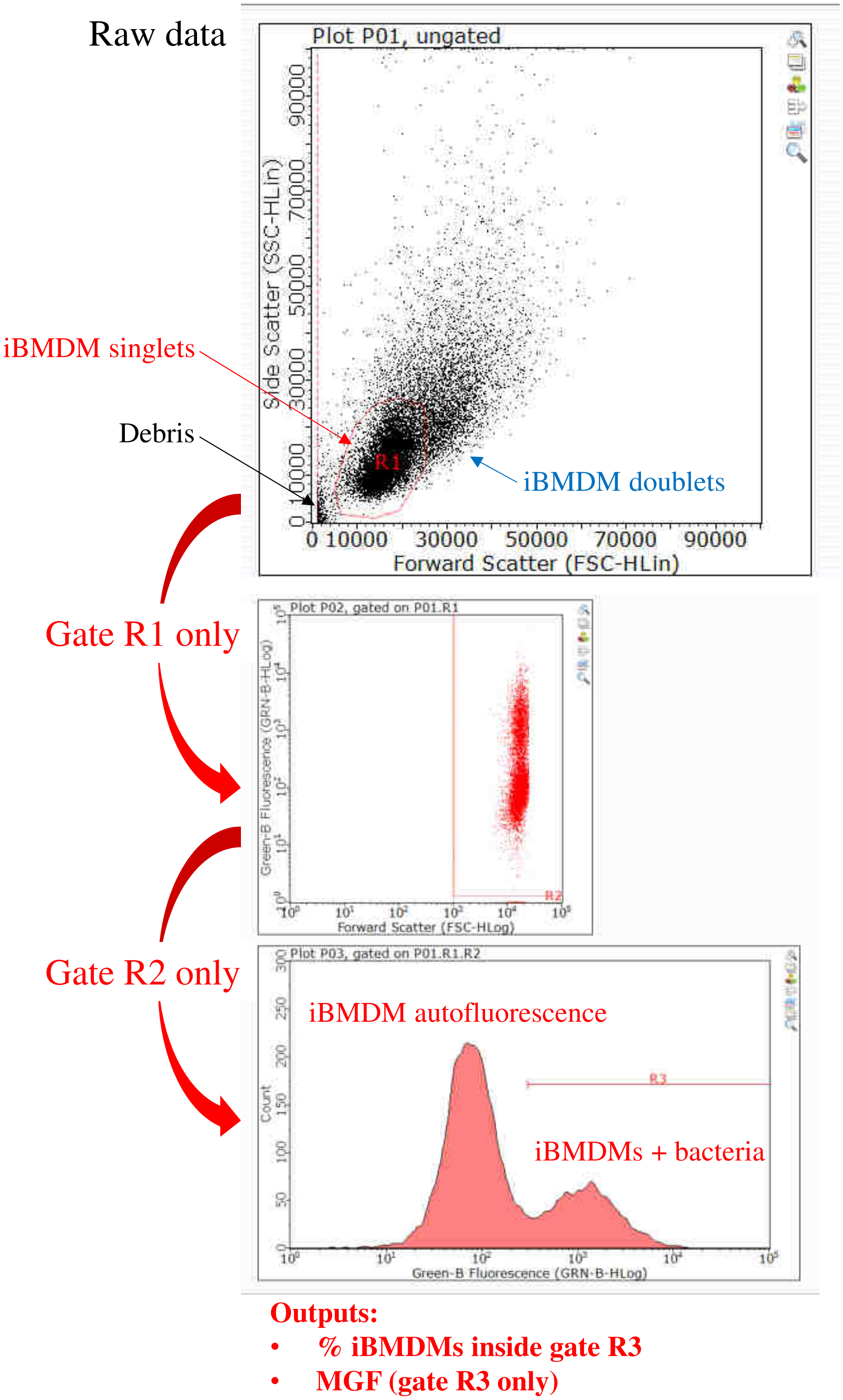


**Fig. S1: Gating strategy for flow cytometry analysis of iBMDMs using GuavaSoft 3.1.1.** Debris and cell clumps were excluded from gate R1. Gate R1 data was re-plotted as FSC log (x axis) versus green fluorescence log (y axis). Gate R2 excluded more debris. Gate R2 data was plotted as a histogram (green fluorescence log (x axis) versus count (y axis)). The left peak (peak green fluorescence ≈ $7 \times 10^1$) corresponds to autofluorescence of empty iBMDMs, whereas the right peak corresponds to iBMDMs with internalised GFP-labelled bacteria. Gate R3 (green fluorescence > $3 \times 10^2$) was drawn to select only the right peak. The percentage of iBMDMs containing bacteria was calculated using (no. iBMDMs in gate R3/no. iBMDMs in gate R2) x 100. The median green fluorescence (MGF) of gate R3 data was calculated by GuavaSoft 3.1.1.

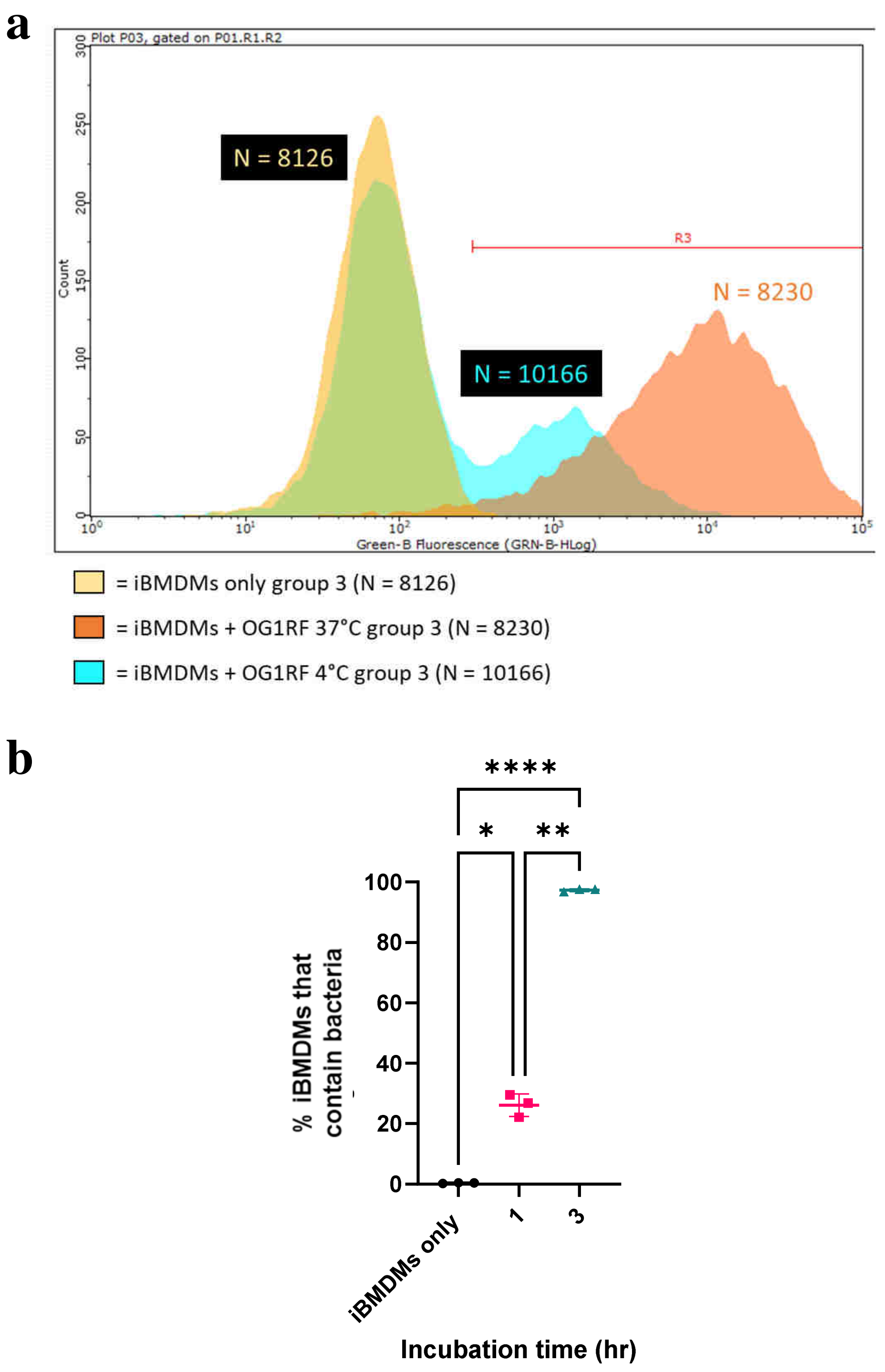


**Fig. S2: Impact of incubation time and multiplicity of Infection (MOI) on *E. faecalis* uptake by iBMDMs. (a)** Histograms plotting green fluorescence of iBMDMs following incubation without treatment (yellow) or with GFP-labelled OG1RF for 1 hr (blue) or 3 hr (orange) at 37°C. The position of gate R3 (which contains bacteria-containing macrophages) is indicated. Each plot represents one of three independent replicates performed for each treatment. In this figure, N = total number of iBMDMs per group. **(b)** Percentage of iBMDMs that contain *E. faecalis* after 1 hour versus 3 hours incubation at 37 °C. A one-way ANOVA with Brown-Forsythe and Welch's correction followed by Dunnett's multiple comparisons test was performed to assess significance. *P*-values: iBMDMs only versus 1 hour, $P = 0.0146$; iBMDMs only versus 3 hours, $P < 0.0001$; 1 hour versus 3 hours, $P = 0.002$. Error bars represent mean ± standard deviation (SD). *P*-value descriptors: *, $P < 0.05$; **, $P < 0.01$; ****, $P < 0.0001$.

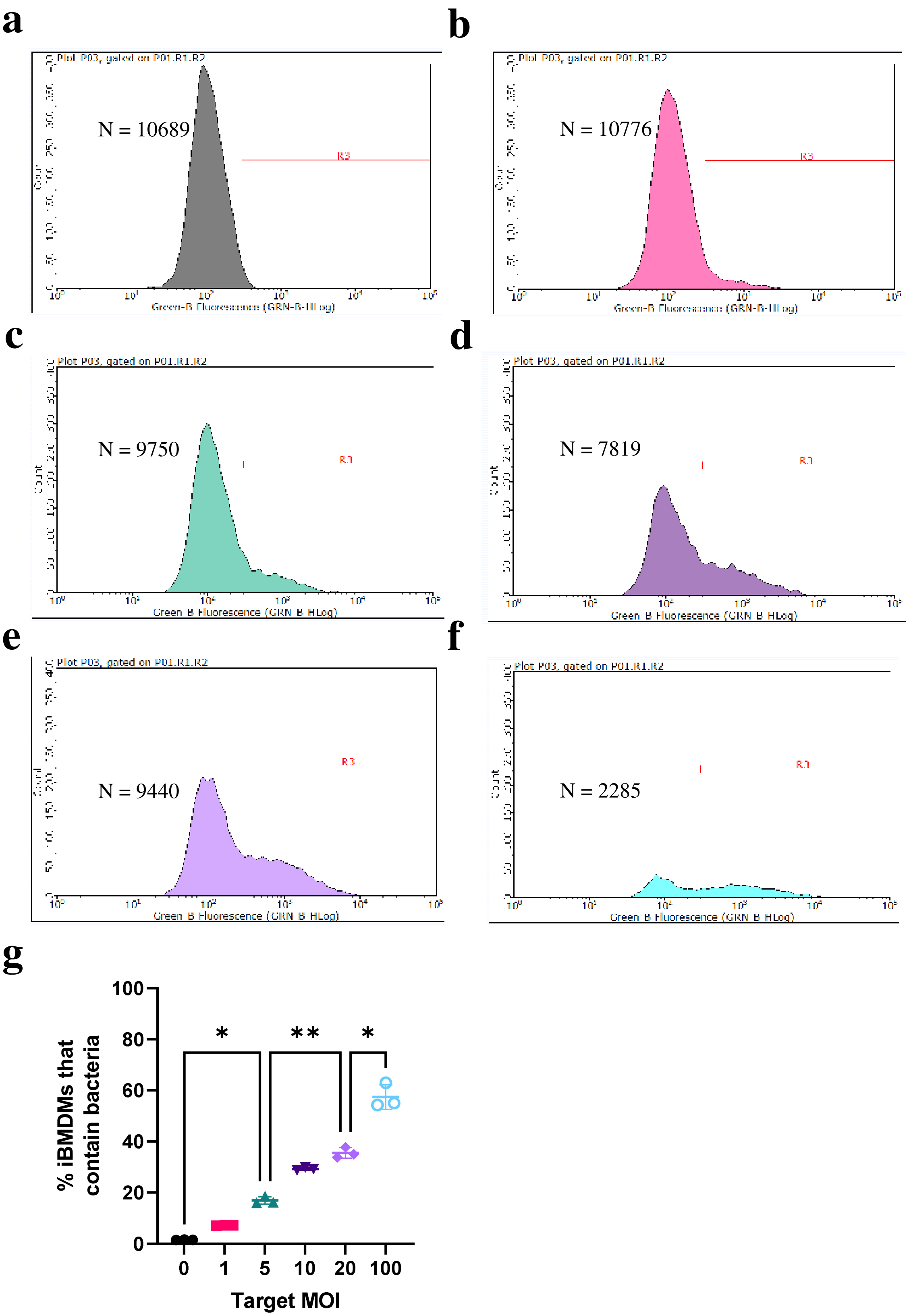


**Fig. S3: Impact of multiplicity of Infection (MOI) on *E. faecalis* uptake by iBMDMs – histograms and proportions. (a-f)** Histograms plotting green fluorescence of iBMDMs following incubation without treatment **(a)** or with GFP-labelled OG1RF at MOI = 1 **(b)**, 5 **(c)**, 10 **(d)**, 20 **(e)**, or 100 **(f)**. On each plot, the position of gate R3 (which contains bacteria-containing macrophages) is indicated. Each plot represents one of three independent replicates performed for each MOI. In this figure, N = total number of iBMDMs per plot. **(g)** Percentage of iBMDMs that contain *E. faecalis* according to bacterial dose (1 hour incubation). Statistical analysis was performed via a one-way ANOVA with Brown-Forsythe and Welch's correction followed by Dunnett's multiple comparisons test. *P*-values: MOI = 0 versus MOI = 5, $P = 0.0126$; MOI = 5 versus MOI = 20, $P = 0.0016$; MOI = 20 versus MOI = 100, $P = 0.0329$. Error bars represent mean ± standard deviation (SD). *P*-value descriptors: *, $P < 0.05$; **, $P < 0.01$.

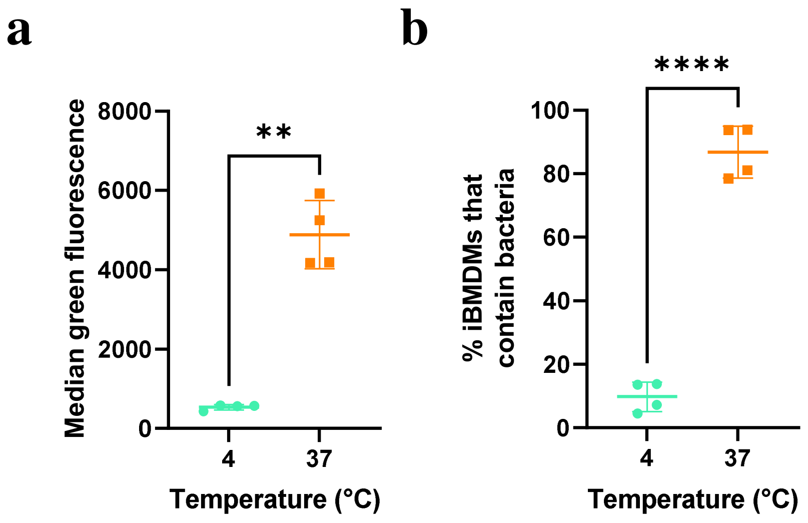


**Fig. S4: Impact of temperature on *E. faecalis* uptake by iBMDMs.** (**a**) *E. faecalis*-positive iBMDMs were significantly more fluorescent at 37 °C as compared to 4 °C. Statistical analysis was performed via an unpaired *t*-test with Welch's correction ($P$ = 0.0019; n = 4 technical replicates). (**b**) The percentage of iBMDMs that contained bacteria was found to be significantly higher at 37 °C as compared to 4 °C. An unpaired *t*-test was performed with Welch's correction ($P$ < 0.0001; n = 4 technical replicates).

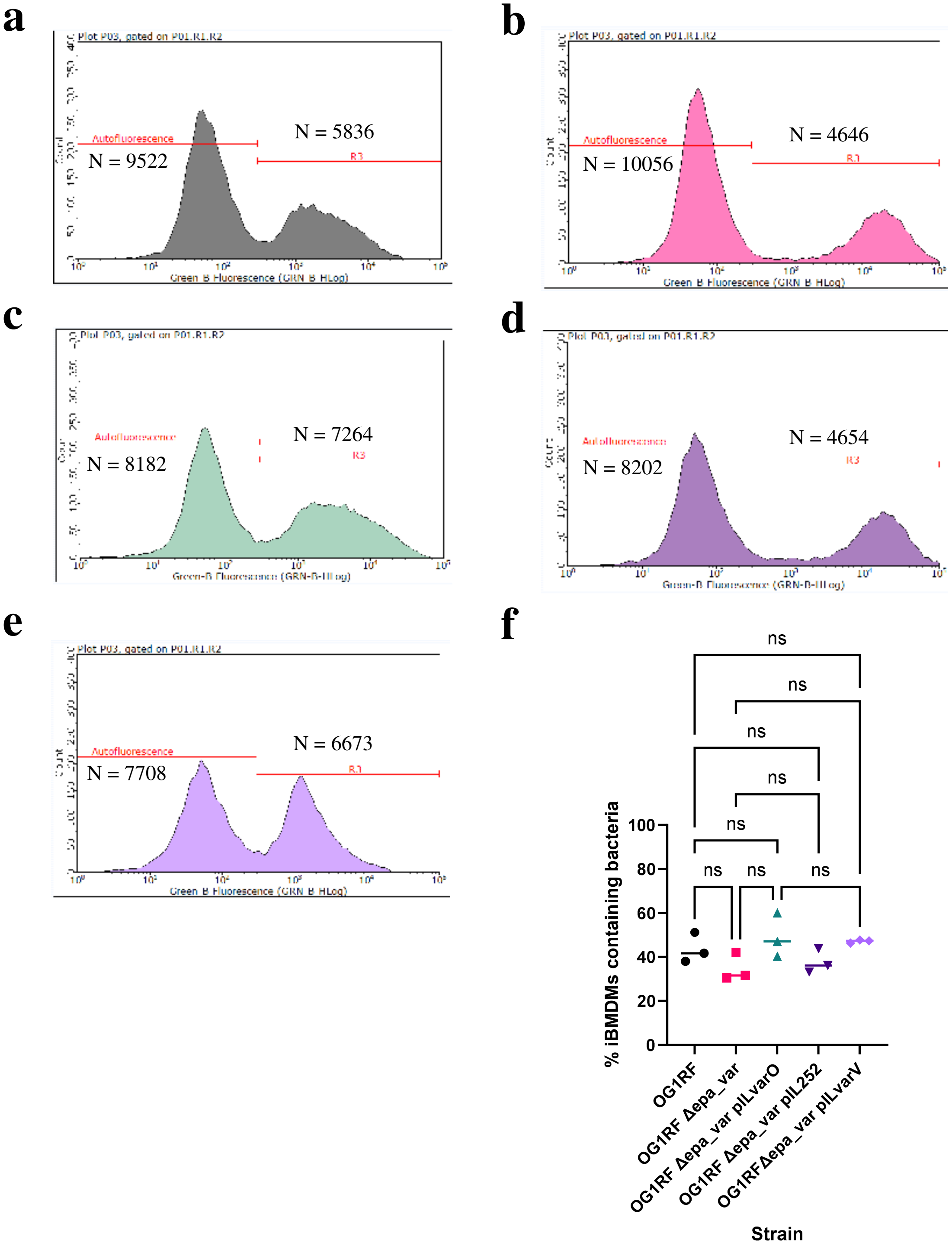


**Fig. S5: Phagocytosis of *E. faecalis* OG1RF derivatives – histograms and proportions. (a-e)** Histograms plotting green fluorescence of iBMDMs following incubation with GFP-labelled OG1RF (**a**), the Δ*epa_var* derivative (**b**), Δ*epa_var* pILvarO (**c**), Δ*epa_var* pIL252 (**d**), or Δ*epa_var* pILvarV (**e**). On each plot, the separation between bacteria-free (Autofluorescence) and bacteria-positive (gate R3) macrophages is indicated. Each plot represents one of three independent replicates performed for each treatment in this experiment. In this figure, N = number of iBMDMs within each gate. (**f**) Percentage of iBMDMs that did contain bacteria. Each value is the mean of three replicates per treatment. Statistical analysis was performed by via one-way ANOVA with Brown-Forsythe and Welch's correction followed by Dunnett's multiple comparisons test. *P*-values: OG1RF versus Δ*epa_var*, $P = 0.664$; OG1RF versus pILvarO, $P = 0.985$; OG1RF versus pIL252, $P = 0.890$; OG1RF versus pILvarV, $P = 0.952$; Δ*epa_var* versus pILvarO, $P = 0.491$; Δ*epa_var* versus pIL252, $P = 0.997$; Δ*epa_var* versus pILvarV, $P = 0.270$; pILvarO versus pILvarV, $P > 0.999$. *P*-value descriptors: ns, not significant.

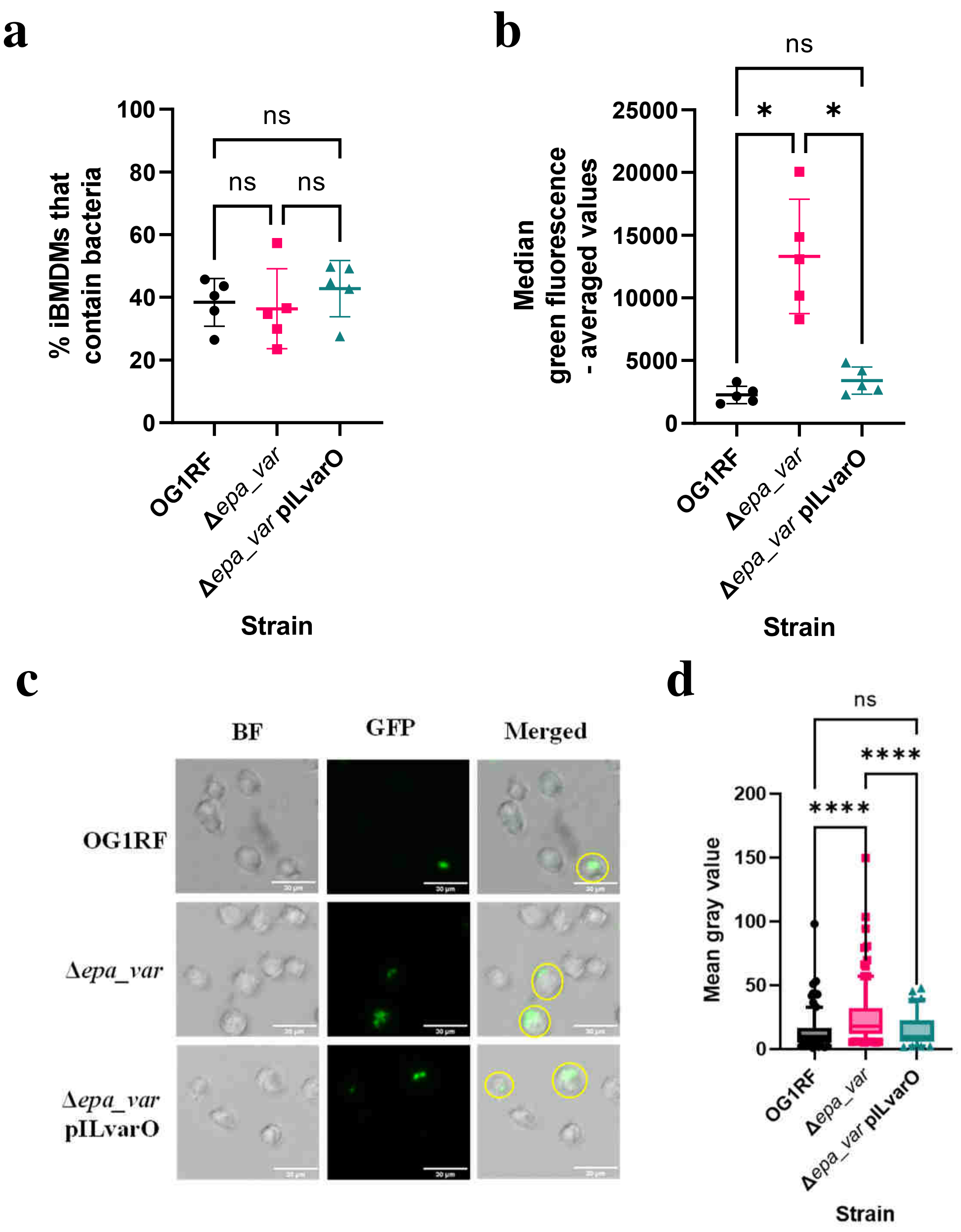


**Fig. S6: Internalisation of *E. faecalis* OG1RF Δ*epa_var* which lacks EPA decorations.** (**a**) Percentage iBMDMs positive for internalised bacteria. *P*-values: OG1RF versus Δ*epa_var*, *P* = 0.985; OG1RF versus pILvarO, *P* = 0.794; Δ*epa_var* versus pILvarO, *P* = 0.743. Statistical analysis was performed by doing a one-way ANOVA with Brown-Forsythe and Welch's correction followed by Dunnett's multiple comparisons test (n = 5 biological replicates per group). (**b**) Green fluorescence intensity of iBMDMs that contained bacteria. *P*-values: OG1RF versus Δ*epa_var*, *P* = 0.0150; OG1RF versus pILvarO, *P* = 0.220; Δ*epa_var* versus pILvarO, *P* = 0.0232. Again, statistical analysis was performed via a one-way ANOVA with Brown-Forsythe and Welch's correction followed by Dunnett's multiple comparisons test (n = 5 biological replicates per group). (**c**) Confocal microscopy of iBMDMs following incubation with GFP-labelled *E. faecalis* – representative images. Yellow circles indicate macrophages analysed in (**d**). BF, brightfield; GFP, GFP channel. (d) Pixel intensity of macrophages with internalised bacteria, measured as mean grey value using ImageJ. All values were normalised by subtracting the mean grey value of the background. Box plots represent medians flanked by upper and lower quartiles ($25^{th}$ and $75^{th}$ percentiles, respectively), while whiskers represent $5^{th}$ and $95^{th}$ percentiles. Statistical analysis was performed by doing a Kruskal-Wallis test followed by Dunn's multiple comparisons test. *P*-values: OG1RF versus Δ*epa_var*, *P* < 0.0001; OG1RF versus pILvarO, *P* > 0.999; Δ*epa_var* versus pILvarO, *P* < 0.0001. Sample sizes: OG1RF, n = 203; Δ*epa_var*, n = 215; Δ*epa_var* pILvarO, n = 98. Key to *P*-values: ns, not significant; *, *P* < 0.05; ****, *P* < 0.0001.

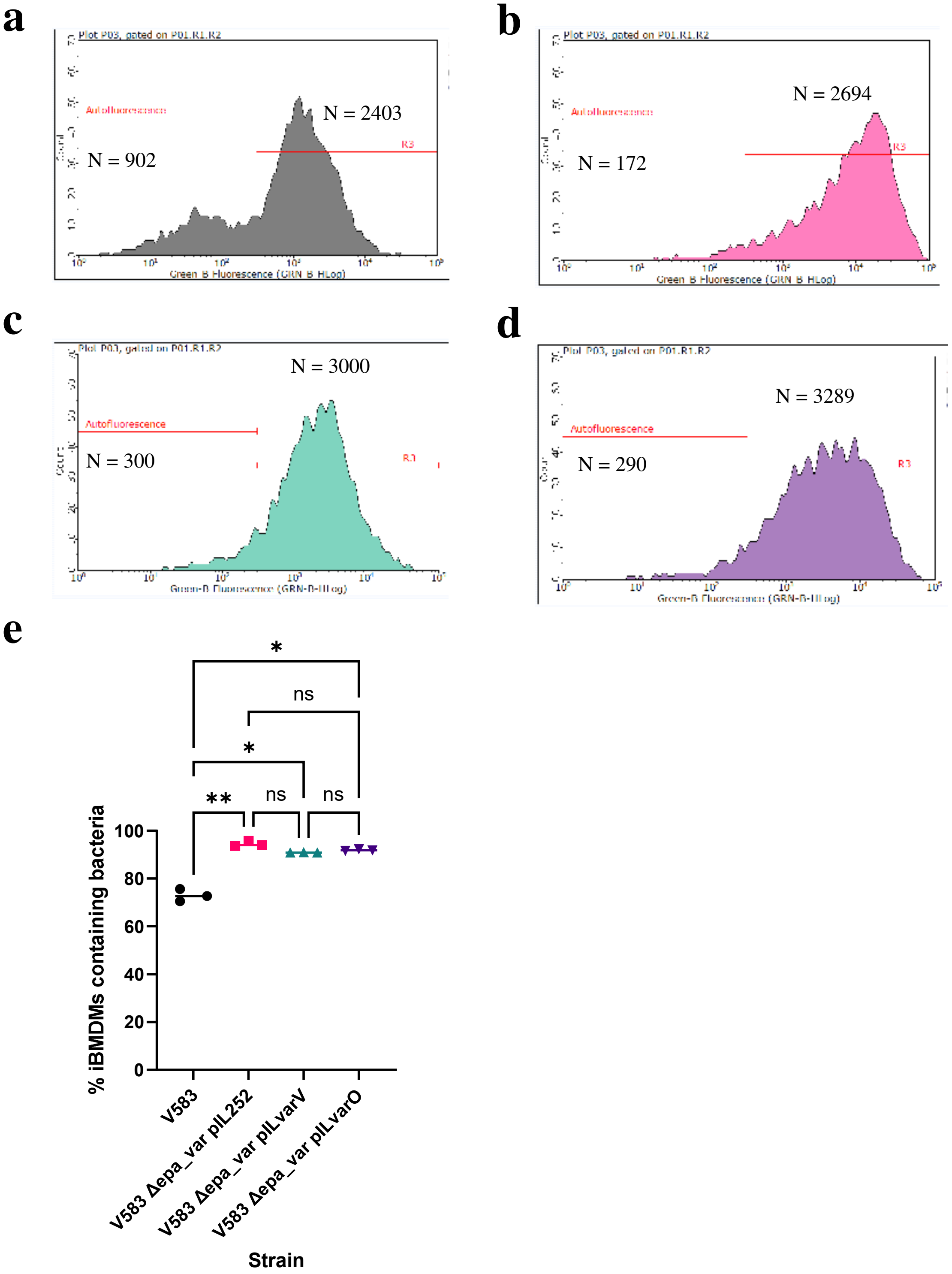


**Fig. S7: Phagocytosis of *E. faecalis* V583 derivatives – histograms and proportions. (a-d)** Histograms plotting green fluorescence of iBMDMs following incubation with GFP-labelled V583 (**a**), or the Δ*epa_var* derivative with pIL252 (**b**), pILvarV (**c**), or pILvarO (**d**). On each plot, the separation between bacteria-free (Autofluorescence) and bacteria-positive (gate R3) macrophages is indicated. Each plot represents one of three independent replicates performed for each treatment in this experiment. In this figure, N = number of iBMDMs within each gate. **(e)** Percentage of iBMDMs that did contain bacteria. Each value is the mean of three replicates per treatment. Statistical analysis was performed by via one-way ANOVA with Brown-Forsythe and Welch's correction followed by Dunnett's multiple comparisons test. *P*-values: V583 versus pIL252, $P = 0.0035$; V583 versus pILvarV, $P = 0.0201$; V583 versus pILvarO, $P = 0.0184$; pIL252 versus pILvarV, $P = 0.103$; pIL252 versus pILvarO, $P = 0.201$; pILvarV versus pILvarO, $P = 0.117$. *P*-value descriptors: ns, not significant; *, $P < 0.05$; **, $P < 0.01$.

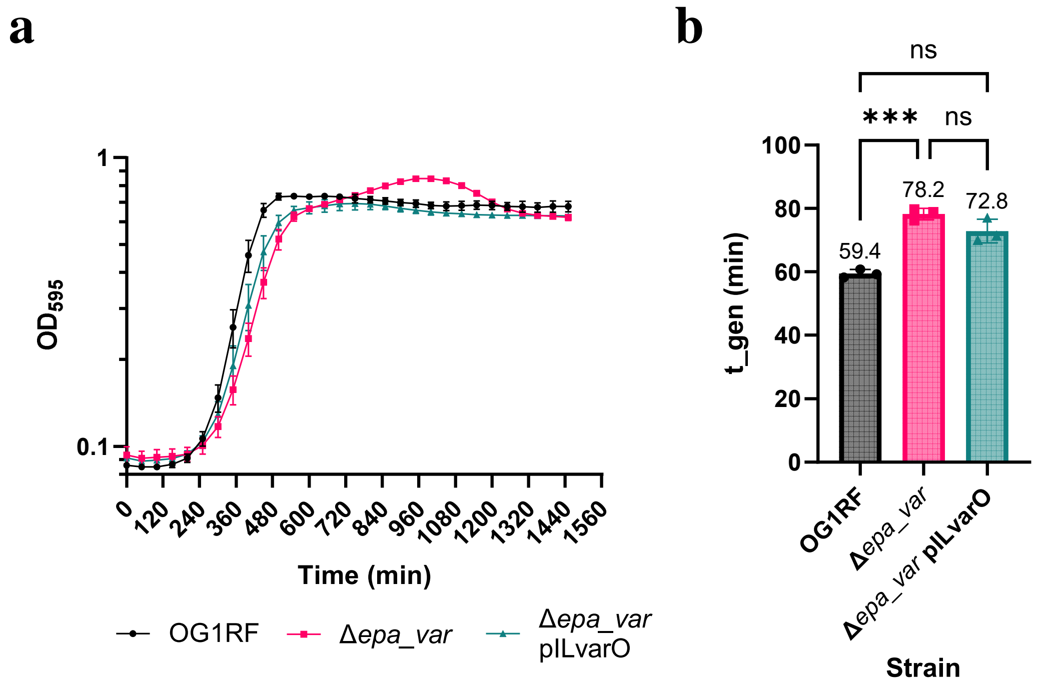


**Fig. S8: Impact of the Δ*epa_var* mutation on *E. faecalis* OG1RF growth rate.** (**a**) Growth profiles of *E. faecalis* OG1RF, Δ*epa_var* and complemented Δ*epa_var* in BHI broth at 37 °C. Each data point represents the mean of three biological replicates ± SD. (**b**) Generation times (t_gen) in min. Three biological replicates per strain were performed. Mean t_gen values were compared via one-way ANOVA with Brown-Forsythe and Welch's correction, followed by Dunnett's multiple comparisons test. *P*-values: OG1RF versus Δ*epa_var*, $P = 0.0003$; OG1RF versus pILvarO, $P = 0.0568$; Δ*epa_var* versus pILvarO, $P = 0.240$. Key to *P*-values: ns, not significant; ***, $P < 0.001$.

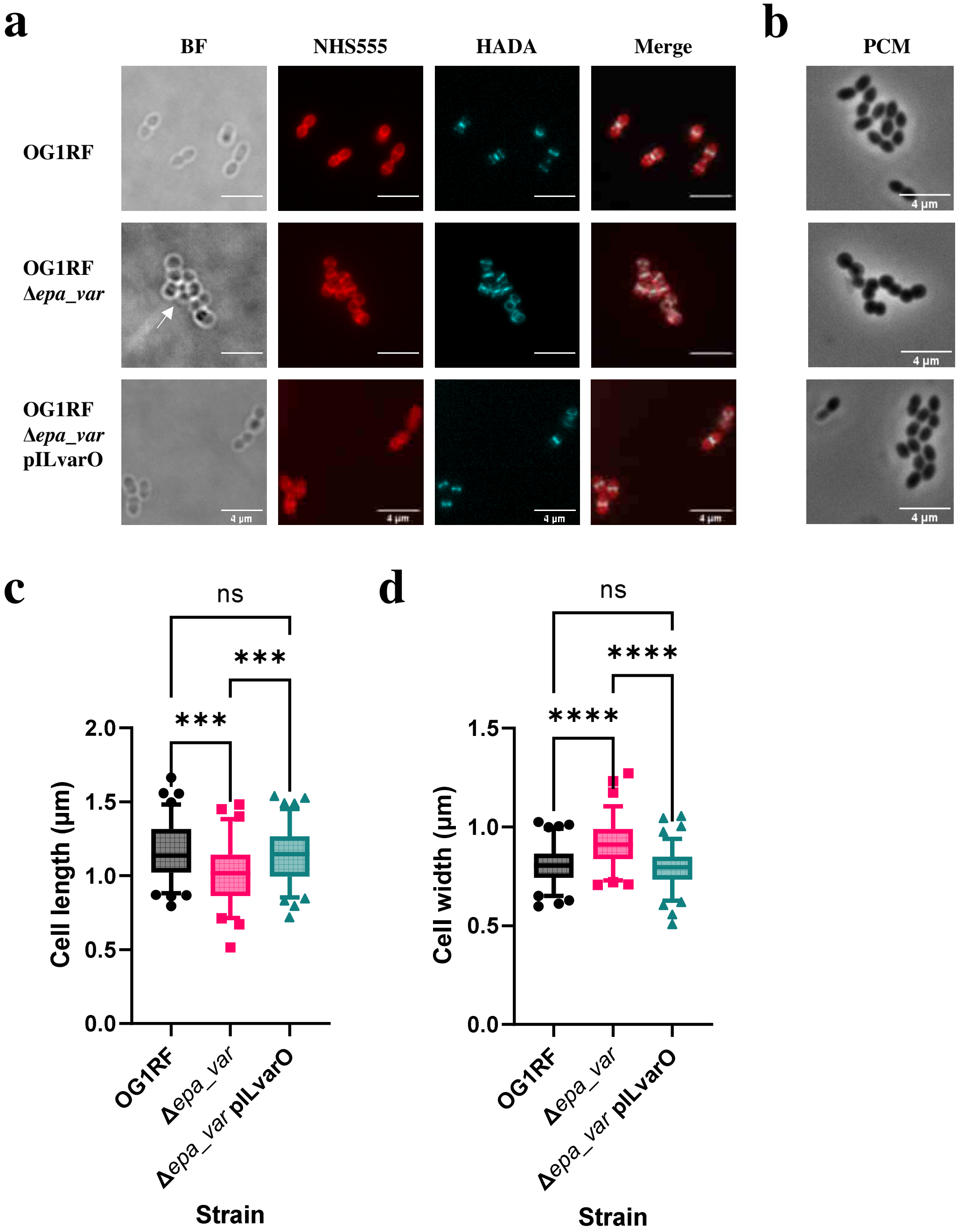


**Fig. S9: Microscopic analysis of *E. faecalis* OG1RF Δ*epa_var* shows an altered morphology of *epa_var*.** (**a**) Fluorescence microscopy of exponential-phase *E. faecalis* bacteria labelled with NHS ester 555 and HADA. White arrows indicate bacterial cell aggregates. All images were taken at 100 x magnification. Scale bar = 4 µm. BF, brightfield. (**b**) Phase contrast microscopy of exponential-phase *E. faecalis*. OG1RF = upper panel; Δ*epa_var* = middle panel; Δ*epa_var* pILvarO = lower panel. Same magnification and scale bar as used in (**a**). (**c**) Comparison of bacterial cell length. Box plots show medians flanked by lower and upper quartiles; whiskers show 5th and 95th percentiles. *P*-values: OG1RF versus Δ*epa_var*, $P = 0.0002$; OG1RF versus pILvarO, $P > 0.999$; Δ*epa_var* versus pILvarO, $P = 0.0002$. Sample sizes: n = 86 (OG1RF); n = 74 (Δ*epa_var*); n = 96 (Δ*epa_var* pILvarO). (**d**) Comparison of bacterial cell width. The same samples were analysed here as (**c**). Box plots show medians flanked by lower and upper quartiles; whiskers show 5th and 95th percentiles. *P*-values: OG1RF versus Δ*epa_var*, $P < 0.0001$; OG1RF versus pILvarO, $P > 0.999$; Δ*epa_var* versus pILvarO, $P < 0.0001$. In both (**c**) and (**d**), statistical comparisons were made by doing a Kruskal-Wallis test, followed by Dunn's multiple comparisons test.

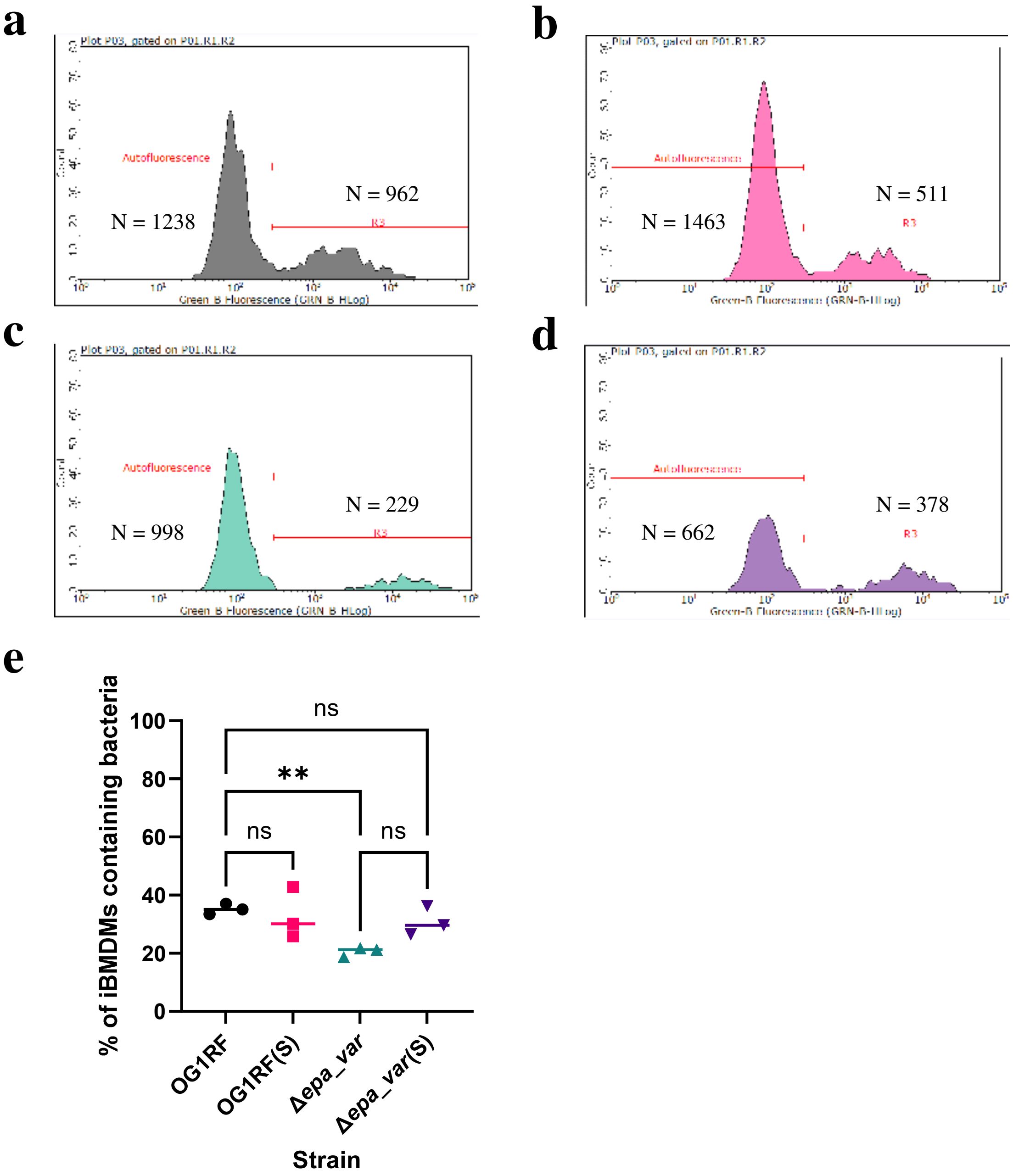


**Fig. S10: Phagocytosis of *E. faecalis* OG1RF or Δ*epa_var* with or without sonication beforehand. (a-d)** Histograms plotting green fluorescence of iBMDMs following incubation with GFP-labelled OG1RF **(a)**, sonicated (S) OG1RF **(b)**, Δ*epa_var* **(c)**, or sonicated (S) Δ*epa_var* bacteria **(d)**. On each plot, the separation between bacteria-free (Autofluorescence) and bacteria-positive (gate R3) macrophages is indicated. Each plot represents one of three independent replicates performed for each treatment in this experiment. In this figure, N = number of iBMDMs within each gate. **(e)** Percentage of iBMDMs that did contain bacteria. Each value is the mean of three replicates per treatment. Statistical analysis was performed using a one-way ANOVA with Brown-Forsythe and Welch's correction followed by Dunnett's multiple comparisons test. *P*-values: OG1RF versus OG1RF(S), $P = 0.995$; OG1RF versus Δ*epa_var*, $P = 0.0022$; OG1RF versus Δ*epa_var*(S), $P = 0.668$; Δ*epa_var* versus Δ*epa_var*(S), $P = 0.224$. Key to *P*-values: ns, not significant; **, $P < 0.01$.

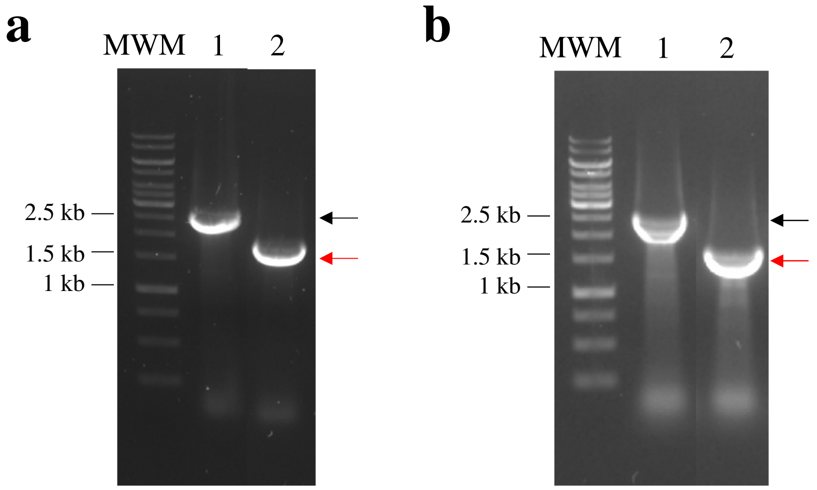


**Figure S11: Gel electrophoresis of colony PCRs to characterize Δ*lgt* in-frame deletion mutants.** Colony PCR using primers SM_0210 and SM_0211 was used to screen Δ*lgt* mutants in the OG1RF wild-type (**a**) and Δ*epa_var* backgrounds (**b**). The expected DNA band sizes corresponding to *lgt* (2,444 bp) and its deleted counterparts (Δ*lgt*, 1,643 bp) are indicated with black and red arrows, respectively. Lane 1, control colony PCR using OG1RF; lane 2, Δ*lgt* mutant.

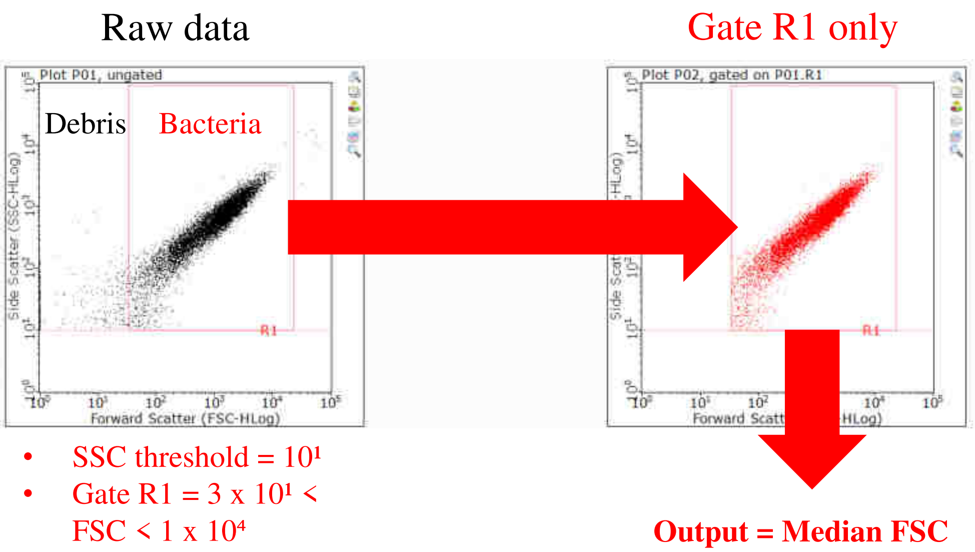


**Figure S12: Flow cytometry gating strategy for *E. faecalis*.** Data points were first plotted as a FSC log (x axis) versus SSC log scatter graph (left panel). Debris were excluded by (i) setting a threshold = $10^1$ for SSC values and (ii) drawing gate R1 spanning 3 x $10^1$ < FSC < 1 x $10^4$ (right panel). Median FSC was determined from all data points within gate R1. FSC, forward scatter; SSC, side scatter.
