# Supplementary material for: Exploring the role of E. faecalis enterococcal polysaccharide antigen (EPA) and lipoproteins in evasion of phagocytosis
Source: Mol Microbiol. Author manuscript; Available in PMC 2025 Sep 9. (PMC7618083; doi:10.1111/mmi.15294)
Supplement: Supplementary Material [file EMS208353-supplement-Supplementary_Material.zip › mmi15294-sup-0002-tables1.pdf]

Table S1: Bacterial strains, plasmids, and oligonucleotides.

Strains/plasmids/ oligonucleotides	Relevant properties/sequence [a]	Source
**Strains**		
Enterococcus faecalis		
OG1RF	Plasmid-free, virulent strain isolated from a human oral cavity	(1)
OG1RF (pGFP)	OG1RF derivative expressing GFP; Tet5	(2)
OG1RF Δ*var*	OG1RF mutant with a complete deletion of the locus encoding EPA decorations including *OG1RF_11720* to *OG1RF_11706*	P. Serror
OG1RF Δ*var* (pGFP)	OG1RF Δ*var* derivative expressing GFP; Tet5	This work
OG1RF Δ*var* (pIL252)	OG1RF Δ*var* derivative harbouring pIL252; Erm3	P. Serror
OG1RF Δ*var* (pIL252, pGFP)	OG1RF Δ*var* (pIL252) derivative expressing GFP; Erm3, Tet5	This work
OG1RF Δ*var* (pIL-O)	OG1RF Δ*var* derivative transformed with pIL-O encoding the EPA decoration locus from *E. faecalis* OG1RF	P. Serror
OG1RF Δ*var* (pIL-O, pGFP)	OG1RF Δ*var* (pIL-O) derivative expressing GFP; Erm3, Tet5	This work
OG1RF Δ*var* (pIL-V)	OG1RF Δ*var* derivative complemented with pIL-V encoding the EPA decoration locus from *E. faecalis* V583	P. Serror
OG1RF Δ*var* (pIL-V, pGFP)	OG1RF Δ*var* (pIL-V) derivative expressing GFP; Erm3, Tet5	This work
OG1RF Δ*lgt*	OG1RF mutant with an in-frame deletion in *lgt*	This work
OG1RF Δ*lgt* (pGFP)	OG1RF Δ*lgt* derivative expressing GFP; Tet5	This work
OG1RF Δ*lgt* (plgt)	OG1RF Δ*lgt* mutant complemented with a plasmid-encoded inducible *lgt* gene	This work
OG1RF Δ*lgt* (plgt, pGFP)	OG1RF Δ*lgt* (pTet_lgt) derivative expressing GFP; Erm30, Tet5	This work
OG1RF Δ*var*Δ*lgt*	OG1RF mutant with an in-frame deletion in *lg*	This work
OG1RF Δ*var*Δ*lgt* (pGFP)	OG1RF Δ*var* derivative with an in-frame deletion in *lgt* expressing GFP; Tet5	This work
OG1RF Δ*var*Δ*lgt* (plgt)	OG1RF Δ*var*Δ*lgt* mutant complemented with a plasmid-encoded inducible *lgt* gene; Erm30	This work
OG1RF Δ*var*Δ*lgt* (plgt, pGFP)	OG1RF Δ*var*Δ*lgt* (plgt) derivative expressing GFP; Erm30, Tet5	This work
V583* (VE18379)	V583 plasmidless derivative	(3)
V583* (pGFP)	V583* derivative expressing GFP; Tet5	This work
V583* Δ*var* (VE18395)	V583* mutant with a complete deletion of the locus encoding EPA decorations including *EF2176* to *EF2164*	(3)
V583* Δ*var* (pIL252) (VE18927)	V583* Δ*var* derivative harbouring pIL252; Erm3	(3)
V583* Δ*var* (pIL252, pGFP)	V583* Δ*var* (pIL252) derivative expressing GFP; Erm3, Tet5	This work
V583* Δ*var* (pIL-V) (VE18930)	V583* Δ*var* derivative complemented with pILvarV encoding the EPA decoration locus from *E. faecalis* V583; Erm3	(3)
V583* Δ*var* (pIL-V, pGFP)	V583* Δ*var* (pILvarV) derivative expressing GFP; Erm3, Tet5	This work
V583* Δ*var* (pIL-O)	V583* Δ*var* derivative complemented with pILvarO encoding the EPA decoration locus from *E. faecalis* OG1RF; Erm3	P. Serror
V583* Δ*var* (pIL-O pGFP)	V583* Δ*var* (pILvarO) derivative expressing GFP; Erm3, Tet5	This work
Escherichia coli		
TG1 (*repA*$^{+}$)	TG1 derivative encoding RepA for pGhost9 propagation at 37°C	(4)
NEB5α	Cloning strain	NEB
**Plasmids**		
pGhost9	Temperature-sensitive plasmid for gene replacement; ErmR	(5)
pG_lgt	pGhost9 derivative used to create an in-frame *lgt* deletion; ErmR	This work
pTetH	pAT18 derivative encoding TetR for tetracycline-inducible expression in *E. faecalis*	(6)

pTetH_lgt	pTetH derivative used to complement the *lgt* mutation	This work
pMV158_GFP	pMV158 derivative consitutively expressing the *gfp*	(7)
pIL252	Low-copy number plasmid used for epa complementation experiements; ErmR	(8)
pILvarO	pIL252 derivative encoding the *epa variable* locus from *E. faecalis* OG1RF (from the 8 last codons of *epaR* to the first 6 codons of *glpQ*)	P. Serror
pILvarV (pVE14388)	pIL252 derivative encoding the complete *epa variable* locus from *E. faecalis* V583	(3)
**Oligonucleotides**		
SM_0100 (pTetH_F)	GCTTGATCGTAGCGTTAACAGATCTACTC	
SM_0101 (pTetH_R)	CAAATTGTGGATGTGACCATGCGG	
SM_0171 (pGhost_F)	GTCACGACGTTGTAAAACGACGG	
SM_0172 (pGhost_R)	CTAGCGGACTCTAGAGGATCCCA	
SM_0194 (pG_lgt_H11)	TATAGGGCGAATTGGGTACCGGGCCCCCCCTCGAGTTTATTAGTGATGCGCCGTTTATGTGC	
SM_0195 (pG_lgt_H12)	TACTTGAGCTAACATCATCACATCTTCTCC	
SM_0196 (pG_lgt_H21)	GATGTGATGATGTTAGCTCAAGTAAAAATAACAACTTCTTAATCAACTTGATCAAAGAATATGG	
SM_0197 (pG_lgt_H22)	CGGACTCTAGAGGATCCCACCGCGGTGGCGGCCGCGCTAAACCACGAGTCATAATTGCCG	
SM_0210 (pG_lgt_H110)	TGACCGTTTACAATTATTTGGGAGC	
SM_0211 (pG_lgt_H220)	TTAAATCCCCAACACCACTTAAACC	
SM_0401 (pTetH_lgt_F)	TCATTGATAGAGTGAGCTCAAGGAGGAGACTGACCATGGAATTAGCTCAAGTAAATTCAATTGC	
SM_0402 (pTetH_lgt_R)	CTTTAGTGATGATGGTGATGGTGATGGTGGGATCCAGAAGTTGTTATTTTTTTCTTTTGTTTCTCCCG	

[a] Erm3, Erythromycin 3µgml^{-1}; Erm30, Erythromycin 30µg ml^{-1}; Tet5, Tetracycline 5µg ml^{-1}

References

(1) Dunny, G. M., Brown, B. L., and Clewell, D. B. (1978) Induced cell aggregation and mating in *Streptococcus faecalis*: evidence for a bacterial sex pheromone. *Proc Natl Acad Sci U S A* **75**, 3479-3483

(2) Prajsnar TK, Renshaw SA, Ogryzko NV, Foster SJ, Serror P, Mesnage S. Zebrafish as a novel vertebrate model to dissect enterococcal pathogenesis. Infect Immun. 2013;81(11):4271-4279

(3) Furlan, S., Matos, R. C., Kennedy, S. P., Doublet, B., Serror, P., and Rigottier-Gois, L. (2019) Fitness restoration of a genetically tractable *Enterococcus faecalis* V583 derivative to study decoration-related phenotypes of the Enterococcal Polysaccharide Antigen. mSphere 4(4), e00310-19

(4) Law, J., Buist, G., Haandrikman, A., Kok, J., Venema, G., and Leenhouts, K. (1995) A system to generate chromosomal mutations in *Lactococcus lactis* which allows fast analysis of targeted genes. *J Bacteriol* **177**, 7011-7018

(5) Maguin E, Duwat P, Hege T, Ehrlich D, Gruss A. New thermosensitive plasmid for gram-positive bacteria. J Bacteriol. 1992;174(17):5633-5638

(6) Salamaga, B., Prajsnar, T. K., Jareno-Martinez, A., Willemse, J., Bewley, M. A., Chau, F., Ben Belkacem, T., Meijer, A. H., Dockrell, D. H., Renshaw, S. A., and Mesnage, S. (2017) Bacterial size matters: Multiple mechanisms controlling septum cleavage and diplococcus formation are critical for the virulence of the opportunistic pathogen *Enterococcus faecalis*. PLoS Pathog 13, e1006526

(7) Nieto, C., and Espinosa, M. (2003) Construction of the mobilizable plasmid pMV158GFP, a derivative of pMV158 that carries the gene encoding the green fluorescent protein. Plasmid 49, 281-285

(8) Simon D, Chopin A (1988) Construction of a vector plasmid family and its use for molecular cloning in *Streptococcus lactis.* Biochimie 70: 559-566

Table S2: Antibiotics used in this study.

Antibiotic	Stock concentration (mg/ml)	Working concentration (µg/ml)	
		E. faecalis	*E. coli*
Ampicillin (Amp)	100	N/A	100
Erythromycin (Erm)	30	30 or 3[a]	200
Gentamicin (Gen)	50	250	N/A
Tetracycline (Tet)	10	5	N/A
Vancomycin (Van)	50	20	**N/A**

[a] Erm concentration is 30µg ml^{-1} for pGhost and 3µg ml^{-1} for pIL252; N/A, not applicable

Table S3: Wavelengths and filters used for microscopy experiments.

Fluorophore	Excitation/emission wavelengths (nm)	Filter
AlexaFluor^{TM} 555 NHS [a] ester	555/572	Texas Red
GFP [b]	488/510	FITC [c]
HADA [d]	395/450	DAPI [e]
Propidium iodide	555/617	Texas Red

[a] NHS, *N*-hydroxy succinimide
[b] GFP, green fluorescent protein
[c] FITC, Fluorescein isothiocyanate
[d] HADA, hydroxycoumarin-carbonyl-amino-D-alanine
[e] DAPI, 4′,6-diamidino-2-phenylindole
